# Effects of anti-pronation shoes on lower limb kinematics and kinetics in female runners with pronated feet: The role of physical fatigue

**DOI:** 10.1371/journal.pone.0216818

**Published:** 2019-05-14

**Authors:** AmirAli Jafarnezhadgero, Seyed Majid Alavi-Mehr, Urs Granacher

**Affiliations:** 1 Department of Physical Education and Sport Sciences, Faculty of Educational Sciences and Psychology, University of Mohaghegh Ardabili, Ardabil, Iran; 2 Division of Training and Movement Sciences, Research Focus Cognition Sciences, University of Potsdam, Potsdam, Germany; James Cook University College of Healthcare Sciences, BRAZIL

## Abstract

Physical fatigue and pronated feet constitute two risk factors for running-related lower limb injuries. Accordingly, different running shoe companies designed anti-pronation shoes with medial support to limit over pronation in runners. However, there is little evidence on the effectiveness and clinical relevance of anti-pronation shoes. This study examined lower limb kinematics and kinetics in young female runners with pronated feet during running with anti-pronation versus regular (neutral) running shoes in unfatigued and fatigued condition. Twenty-six female runners aged 24.1±5.6 years with pronated feet volunteered to participate in this study. Kinetic (3D Kistler force plate) and kinematic analyses (Vicon motion analysis system) were conducted to record participants’ ground reaction forces and joint kinematics when running with anti-pronation compared with neutral running shoes. Physical fatigue was induced through an individualized submaximal running protocol on a motorized treadmill using rate of perceived exertion and heart rate monitoring. The statistical analyses indicated significant main effects of “footwear” for peak ankle inversion, peak ankle eversion, and peak hip internal rotation angles (p<0.03; d = 0.46–0.95). Pair-wise comparisons revealed a significantly greater peak ankle inversion angle (p<0.03; d = 0.95; 2.70°) and smaller peak eversion angle (p<0.03; d = 0.46; 2.53°) when running with anti-pronation shoes compared with neutral shoes. For kinetic data, significant main effects of “footwear” were found for peak ankle dorsiflexor moment, peak knee extensor moment, peak hip flexor moment, peak hip extensor moment, peak hip abductor moment, and peak hip internal rotator moment (p<0.02; d = 1.00–1.79). For peak positive hip power in sagittal and frontal planes and peak negative hip power in horizontal plane, we observed significant main effects of “footwear” (p<0.03; d = 0.92–1.06). Pairwise comparisons revealed that peak positive hip power in sagittal plane (p<0.03; d = 0.98; 2.39 w/kg), peak positive hip power in frontal plane (p = 0.014; d = 1.06; 0.54 w/kg), and peak negative hip power in horizontal plane (p<0.03; d = 0.92; 0.43 w/kg) were greater with anti-pronation shoes. Furthermore, the statistical analyses indicated significant main effects of “Fatigue” for peak ankle inversion, peak ankle eversion, and peak knee external rotation angles. Pair-wise comparisons revealed a fatigue-induced decrease in peak ankle inversion angle (p<0.01; d = 1.23; 2.69°) and a fatigue-induced increase in peak knee external rotation angle (p<0.05; d = 0.83; 5.40°). In addition, a fatigue-related increase was found for peak ankle eversion (p<0.01; d = 1.24; 2.67°). For kinetic data, we observed a significant main effect of “Fatigue” for knee flexor moment, knee internal rotator moment, and hip extensor moment (p<0.05; d = 0.83–1.01). The statistical analyses indicated significant a main effect of “Fatigue” for peak negative ankle power in sagittal plane (p<0.01; d = 1.25). Finally, we could not detect any significant footwear by fatigue interaction effects for all measures of joint kinetics and kinematics. Running in anti-pronation compared with neutral running shoes produced lower peak moments and powers in lower limb joints and better control in rear foot eversion. Physical fatigue increased peak moments and powers in lower limb joints irrespective of the type of footwear.

## Introduction

High-mileage or high-intensity running result in physical fatigue and subsequent performance decrements. Fatigue-related performance declines develop due to peripheral changes at the level of the muscle and/or due to insufficient drive of the central nervous system to the motor neurons [1]. As physical fatigue sets in, running technique deteriorates and altered lower limb kinetics and kinematics emerge [2]. For instance in long-distance running, fatigue induces changes in lower limb kinematics (e.g., increase in maximal knee extension angle) which results in altered running mechanics in the form of increased loading under the medial arch of the foot [3]. In addition, Mizrahi et al. (2000) showed that running-related fatigue induced an attenuation of the impact accelerations between the tibial tuberosity and sacrum levels [4]. In another study, it was shown that running-related fatigue resulted in increased dorsiflexion velocity, decreased maximum plantar flexion moment, increased knee internal rotation excursion, and decreased knee flexion moment during running [5]. Altered running mechanics have a negative impact on lower limb loading, thus increasing the risk of sustaining running-related acute and/or overuse injuries (RRI) [6]. It has been reported RRI incidence rates which ranged from 19.4–79.3% [7]. The most commonly reported factor associated with RRI is previous injury [8]. Another of the most commonly reported factors associated with RRI is physical fatigue [7].

Besides physical fatigue, overpronation of the feet results in altered lower limb alignment and may therefore constitute another risk factor for RRI [9]. Throughout the running and walking cycle, excessive foot pronation results in excessive internal tibia rotation [10, 11], excessive internal hip rotation [10], anterior pelvis tilt or ipsilateral pelvis drop [11], and altered alignments of the lumbar spine [11]. Common RRI include medial tibial stress syndrome [12], achilles tendinopathy [13], patellofemoral disorders [14], and low back pain [10]. However, it should be noted that there is no consensus in the literature on the effects of overpronation on RRI [15].

Taken together, it seems that excessive overpronation of the feet in the form of altered rearfoot angle together with physical fatigue constitute two major risk factors for RRI [16]. For this purpose, different running shoe companies developed anti-pronation shoes with medial support to limit excessive foot motions in runners, particularly in fatigued condition. Notably, anti-pronation shoes include a reinforced heel counter and a denser midsole to help control any excessive foot pronation. Yet, the effectiveness of anti-pronation shoes on lower limb kinematics (i.e., knee and hip joints) and kinetics is not well understood [16]. In one of the few available studies, Clarke et al. [17] found that shoes with a medial support compared with shoes with a softer midsole significantly reduced pronation and total rearfoot movement. Other studies reported reduced tibial internal rotation when running in anti-pronation shoes [18, 19]. Despite the evidence for the effectiveness of anti-pronation shoes on lower limb kinematics and kinetics, it is somewhat surprising that these changes did not translate to a reduction in number of running related injuries [20]. It is noteworthy that fatigue may play a moderating role as to the effectiveness of anti-pronation shoes on lower limb alignment and ultimately injury rates. There is however no study available that examined the interaction effect of anti-pronation shoes with fatigue on three-dimensional lower limb (knee and hip joints) kinematics and kinetics (joint moments and power).

Therefore, this study was initiated due to scarce information on the effects of anti-pronation shoes on lower limb kinematics and kinetics during running, particularly in fatigued condition. Thus, the main objectives of this study were (i) to examine lower limb joint angles, moments, and powers during running with anti-pronation shoes compared with neutral shoes, and (ii) to elucidate how physical fatigue affects lower limb joint moments and powers when running in anti-pronation shoes compared with neutral footwear. In accordance with the relevant literature [18, 19], we hypothesized that anti-pronation shoes with medial support provide better foot stability and lower limb alignment in unfatigued and particularly fatigued condition.

## Materials and methods

### Participants

This study was conducted at the University of Mohaghegh Ardabili, Iran. The participants were recruited from local sport gyms and clubs. Twenty-six female recreational runners with excessive foot pronation (age: 24.1±5.6 years; height: 165.5±10.2 cm; body mass: 64.2±12.1 kg) and no other diagnosed neural, musculoskeletal injuries or cardiopulmonary diseases during the six months prior to the start of the study volunteered to participate in this study. Previous studies reported differences in biomechanical walking and running characteristics between females and males [21]. More specifically, it has been shown that women compared with men walked with greater transverse plane pelvis and torso rotation, greater hip ab/adduction, hip rotation, knee abduction as well as greater ankle flexion/extension [21]. Based on these findings, female runners were recruited for this study.

Participants were included in this study if they (i) had excessive pronation, as defined by previous studies [22], (ii) showed a heel-strike pattern during running, and (iii) regularly exercised (running) for 2 to 3 times per week over the past 3 years with a single session duration of 45 min. Previous studies demonstrated that runners gain more shock absorption (lower collision force), lower patellofemoral joint stress, and higher muscle activity in the gastrocnemius if they change to a forefoot striking pattern [23]. However, forefoot strike is considered the less efficient running pattern because the center of pressure trajectory is directed posterior after landing and subsequently moves anterior. Runners with a heel strike pattern show a center of pressure trajectory after landing that immediately points in anterior direction [24]. A participant’s foot was considered overpronated if there was a navicular drop >10 mm and a foot posture index (FPI) of greater than 10 [22]. FPI consists of six items that are used to quantify and classify foot posture [22, 25]. These are (i) palpation of the head of the talus; (ii) curvatures above and below the lateral malleolus; (iii) position of the calcaneus in the frontal plane; (iv) prominence in the talonavicular joint; (v) the medial longitudinal arch's congruence; and (vi) abduction/adduction of the forefoot. Each item was rated on a visual analogue scale ranging from −2 to 2, resulting in a total score from −12 to 12. Negative values indicate supinated foot posture and positive values indicate pronated foot posture. The detailed description of the FPI can be found elsewhere [25]. Exclusion criteria were training for any competitive races during the intervention period. Prior to study participation, written informed consent was obtained from all participants. Ethics approval was provided from the Research Ethics Board of the Medical Sciences, University of Ardabil (IR-ARUMS-REC-1396-135), and registered with the Iranian Registry of Clinical Trials (IRCT20170806035517N2; URL: https://www.irct.ir/user/trial/30347/view).

### Experimental design

Participants attended two experimental sessions in our biomechanical laboratory which were separated by at least seven days to allow sufficient recovery. On each testing day, participants were asked to run while wearing either anti-pronation shoes designed for over-pronators (ASICS Women's GEL-Kayano 24 Running Shoe) or neutral running shoes (ASICS Women's GEL-Nimbus 19 Running Shoe). The order of the running shoes across the test days was randomized. These shoe models were selected based on their availability in the local market and their comparable design. According to the manufacturer website (https://www.asics.com/us/en-us/gel-kayano-24/p/0010298530.9016; https://www.asics.com/us/en-us/gel-nimbus-19/p/0010291326.9701), main characteristics of both running shoes were a heel height of 25 mm, a forefoot height of 12 mm, and a heel to toe drop of 13 mm. The manufacturer used FlyteFoam in the midsole of both shoe types. In addition, rearfoot and forefoot GEL Technology is included in the midsole of the anti-pronation shoe to provide better protection against impact. In both shoes, the Dynamic DuoMax Support System ensures stability. DuoMax works with the I.G.S. (IMPACT GUIDANCE SYSTEM) and Guidance Trusstic System which supports heel-to-toe transition by supporting the forefoot and the rear foot. The outsoles of both shoes contain two proprietary rubber compounds that work together in order to deliver traction and responsible protection against wear and tear. According to the manufacturer, the major structural difference between the two selected shoes was the composite materials in the midsole. This information was received through email communication. Prior to the study, participants were also running in New Balance shoes. However, they did not wear anti-pronation shoes.

Upon entering the laboratory, a specific set of retro-reflexive markers were placed on the participants’ specific anatomical landmarks and segments. Thereafter, a baseline assessment was conducted in our laboratory using force plates and a motion capturing system (Vicon) to record kinematics during running at ~3.3 m/s [2]. Subsequently, participants performed a fatigue protocol while running on a motorized treadmill (Quinton Cardiology Inc., Bothell, WA). The fatiguing protocol was terminated after two minutes when a participant reported perceived exertion equal or above 17, or if the individual’s heart rate was above 80% of the maximum. Finally, the baseline running protocol was repeated immediately after completion of the fatigue protocol.

### Pre- and Post-fatigue running kinematics

Ground reaction forces (Fx, Fy and Fz) and moments (Mx, My and Mz) were recorded using two force plates (Kistler AG, Winterthur, Switzerland) embedded in the middle of an 18-m walkway. These force plates (sampling rate of 1000 Hz) were connected to a Vicon MX system (Vicon Motion Systems, Oxford, UK) that recorded GRF data. Participants were familiarized with the laboratory environment and runway area and at least five practice trials were performed. Prior to data collection, participants were asked to run for 10 minutes with each shoe to allow short-term adaptation and familiarization with the respective shoe type. The sequence of shoe wearing was randomized. By doing so, we ensured that participants were able to strike the force plate without consciously changing their running cadence. Afterwards, each participant identified with heel strike pattern during running (kinematic analysis) performed five acceptable running trials at a given running speed of 3.3 m/s. From a biomechanical viewpoint, there are differences between different striking patterns of running [23]. Accordingly, only runners with heel strike patterns were selected for this study. A 3D motion analysis system (Vicon Nexus, Oxford Metrics, UK) was used to record the spatial position of markers on the relevant body segments. The sampling rate was 200 Hz. Three complete force plate strikes for each foot were registered. Before motion capturing started, selected anthropometrical parameters (e.g., height, mass, pelvic width, knee width, ankle width, leg length etc) were assessed and entered into the Nexus software. All reflective markers were placed directly on the skin of the relevant anatomical landmarks and not on the shoe. This allowed a precise tracking of the markers during the running trials. Of note, previous studies have shown that the placement of calcaneus markers on the shoe overestimate rearfoot motion [26]. The CAST marker set technique [27] was employed whereby rigid clusters of four non orthogonal markers were positioned over the lateral shank and lateral thigh to track the segmental kinematics in six degrees of freedom. The overall number of markers amounted to 22. Anatomical markers were placed over the iliac crests, L5-S1 joint, greater trochanter, medial and lateral femoral epicondyles, medial and lateral malleoli, first and fifth metatarsophalangeal joints, and the most anterior part of the toe. Segmental tracking markers located on rigid clusters were placed over the distal portions of the shank and thigh, as well as individual markers located on the superior, inferior and lateral aspects of the heel counter of the heel. A static calibration trial was recorded with all retro-reflective markers (anatomical and tracking) placed on the skeleton and was used to establish joint centers and segment coordinate systems. Anatomical markers were removed following the standing calibration trial. Thereafter, dynamic data collection started. The centers of rotation for the knee and ankle joints were defined statically as the midpoint between the medial and lateral femoral condyle and malleolus markers [28]. The center of the hip joint was calculated using a geometrical prediction method [28]. The major trochanter marker was used to improve the prediction of the hip joint center by immediately calculating the distance between the anterior superior iliac spine and the major trochanter using anatomical landmarks [28]. In visual 3D (C-Motion, Rockville, Maryland), joint kinematics were calculated using an X-Y-Z Euler rotation sequence equivalent to the joint coordinate system and joint kinetic data were calculated using three-dimensional inverse dynamics. Recommendations provided by the International Society of Biomechanics were used to define participants’ rear foot inversion/eversion angles [29]. In this study, inertial parameters were estimated from established anthropometric data [30]. Lower limb joint moments and powers were normalized to each participant's body mass (Nm/kg and w/kg, respectively). Speed was monitored using optical timing gates. A trial was discarded if the dominant foot did not land on the force plates, if the participant targeted the platforms, lost balance during the trial, ran with mid or forefoot strike pattern, or even fell during running. A 10 cm visual analog scale was used to assess the level of comfort of the footwear. Participants were asked to mark their responses on a 10 cm visual analog scale after each test condition.

### Fatigue protocol on the treadmill

The fatigue protocol consisted of running on a motorized treadmill with no inclination (Horizon Fitness, Omega GT, USA), while heart rate was monitored continuously (Polar RS100, Polar Electro Oy, Woodbury, NY). Participants started the test at 6 km/h, and the treadmill speed was increased 1 km/h every 2 minutes. The perceived exertion was collected from participants at the end of each stage using a 15-point Borg scale [31]. As soon as participants reported a perceived exertion of 13 or higher, the treadmill speed was fixed to allow for steady-state running. Throughout this steady-state period, perceived exertion and heart rate were collected every 30 seconds. Maximum heart rate was determined using the equation 220-age [32]. The fatigue protocol was terminated after a two minute steady state run beyond 17 on the Borg scale or ≥80% of the maximum heart rate [33]. Furthermore, blood lactate was measured using a calibrated Accutrend Lactate analyzer (Roche, Mannheim, DL). For this purpose, we inserted a 20 gauge catheter into the antecubital vein. The treatment of blood samples through catheters followed sterile and standard procedures: (i) draw 0.5 mL blood to clear catheter and discard, (ii) draw 3 mL blood and (iii) inject 1 mL sterile saline to keep catheter clean. Thereafter, participants rested quietly for 5 min prior to the physical fatigue protocol. After completion of the physical fatigue protocol, another 3 mL blood sample was taken. The blood sample was immediately tested for lactate concentration.

### Data analyses

Kinematic and kinetic data were analyzed during the stance phase of running which was defined as the interval from ground contact (onset of vertical GRF [Fz] >10 N) to toe off (vertical GRF [Fz] <10 N). Kinematic and kinetic data were filtered using a fourth-order low-pass Butterworth filter with a cutoff frequency of 10 and 20 Hz, respectively. Subsequently, spline interpolation was used to normalize GRF data to 100% of the stance period. All joint moments and power values were normalized with respect to each individual’s body mass (kg).

### Statistical analyses

Data are presented as group mean values and standard deviations. After normal distribution was examined and confirmed using the Shapiro-Wilk-Test, a separate 2 (fatigue: Pre *vs* Post) × 2 (footwear: anti-pronation *vs* neutral shoe) ANOVA with repeated measures was used for statistical analysis. Group-specific and Bonferroni corrected pre-post changes were calculated with the help of paired sample t-tests. Additionally, effect sizes were determined by converting partial eta-squared (η^2^_p_) to Cohen’s d. According to Cohen [34], *d* < 0.50 indicate small effects, 0.50 ≤ *d<* 0.80 indicate medium effects, and *d* ≥ 0.80 indicate large effects. The significance level was set at *p* < 0.05. All analyses were performed using Statistical Package for Social Sciences (SPSS) version 22.0.

## Results

No significant differences were observed for shoe comfort between the neutral (5.4±2.7) and the anti-pronation shoe (5.8±2.9) (p>0.05). Furthermore, running speed was not significantly different (p>0.05) between the four experimental conditions (neutral shoe unfatigued: 3.29±0.03 m/s; neutral shoe fatigued: 3.29±0.04 m/s; anti-pronation shoe unfatigued: 3.30±0.03; anti-pronation shoe fatigued: 3.29±0.04). Significant fatigue-related increases in blood lactate were found for the neutral shoe (13.0±1.6 to 78.4±8.5 mmol/l, p<0.001) and the anti-pronation shoe (13.2±1.7 to 77.7±9.2 mmol/L, p<0.001). No significant differences were observed for blood lactate between running in the neutral (change: 65.4±8.4 mmol/l) versus the anti-pronation shoe (change: 64.5±8.9 mmol/L) (p>0.05). The average time in steady state running during the fatigue protocol was similar for neutral shoe (11.0±3.3 min) and anti-pronation condition (11.2±3.2 min) (p>0.05). Moreover, the average HR within the last two minutes of the fatigue condition was similar for the neutral shoe (177.6±7.4 beats/min) and the anti-pronation shoe (178.1±7.6 beats/min) (p>0.05). Finally, no significant difference was found for average RPE within the last two minutes of the fatigue protocol for neutral and anti-pronation shoe condition (18.1±0.8 vs 18.0±0.8; p>0.05).

The statistical analyses indicated significant main effects of “footwear” for peak ankle inversion, peak ankle eversion, and peak hip internal rotation angles (p<0.03; d = 0.46–0.95) (Table 1). Pair-wise comparisons revealed a significantly greater peak ankle inversion angle (p<0.03; d = 0.95; 2.70°) and smaller peak eversion angle (p<0.03; d = 0.46; 2.53°) when running with anti-pronation shoes compared with neutral shoes.

**Table 1:** Data are means and standard deviations for footwear-specific peak joint angles (degrees) in sagittal, frontal and horizontal planes during running in fatigued and unfatigued condition.

Joint	Angle (degree)	Neutral shoe				Anti-pronation shoe				Footwear	Fatigue	Footwear x Fatigue
		Pre	Post	Δ	95% CI	Pre	Post	Δ	95% CI			
Ankle												
	Inversion	7.98±6.38	4.06±6.58	-3.92	0.61; 7.21	9.45±8.83	7.99±7.32	-1.46	-1.76; 4.69	0.026 (0.947)	0.005 (1.229)	0.362 (0.369)
	Eversion	6.78±7.49	8.31±8.64	1.53	-4.69; 1.63	3.10±7.07	6.90±6.18	3.8	-7.17; -0.43	0.029 (0.462)	0.005 (1.241)	0.401 (0.339)
Knee												
	Internal rotation	6.98±13.62	11.40±13.34	4.42	2.52; -11.36	5.13±11.14	10.54±14.82		1.93; -12.76	0.524 (0.265)	0.089 (0.707)	0.813 (0.090)
	External rotation	12.54±14.57	17.85±11.78	5.31	1.89; -12.50	12.19±12.86	17.68±14.60	5.41	1.57; -12.56	0.900 (0.063)	0.048 (0.834)	0.968 (0.000)
Hip												
	Internal rotation	15.63±9.64	14.90±16.30	-0.73	-4.93; 6.39	14.25±10.93	12.22±14.57	-2.03	-3.94; 7.99	0.210 (0.514)	0.602 (0.211)	0.561 (0.238)
	External rotation	9.53±9.55	7.10±13.50	-2.43	-2.87; 7.74	7.10±9.45	3.51±14.65	-3.59	-2.74; 9.90	0.036 (0.886)	0.191 (0.536)	0.743 (0.127)

Also, the statistical analyses indicated significant main effects of “Fatigue” for peak ankle inversion, peak ankle eversion, and peak knee external rotation angles (p<0.05; d = 0.83–1.24) (Table 1). Pair-wise comparisons revealed a fatigue-related decrease in peak ankle inversion (p<0.01; d = 1.23; 2.69°) and a fatigue-related increase in peak knee external rotation angles (p<0.05; d = 0.83; 5.40°). A significant increase was found for peak ankle eversion (p<0.01; d = 1.24; 2.67°). Moreover, the statistical analysis did not yield any significant footwear by fatigue interactions for joint angles (Table 1).

For kinetic data, significant main effects of “footwear” were found for peak ankle dorsiflexor moment, peak knee extensor moment, peak hip flexor moment, peak hip extensor moment, peak hip abductor moment, and peak hip internal rotator moment (p<0.02; d = 1.00–1.79) (Table 2). Pair-wise comparisons revealed significantly lower peak ankle dorsiflexor moments (p<0.01; d = 1.35; 0.04 N.m/kg) and peak knee extensor moments (p<0.01; d = 1.25; 0.27 N.m/kg) when running with anti-pronation shoes compared with neutral shoes. Moreover, peak hip flexor moment (p<0.001; d = 1.79; 0.72 N.m/kg) was higher when running in neutral shoes compared with anti-pronation shoes. In addition, peak hip extensor moment (p<0.02; d = 1.00; 1.26 N.m/kg) was lower when running in neutral shoes compared with anti-pronation shoes.

**Table 2:** Data are means and standard deviations for footwear-specific peak joint moments (N.m/kg) in sagittal, frontal and horizontal planes during running in fatigued and unfatigued condition.

Joint	Moment (N.m/kg)	Neutral shoe				Anti-pronation shoe				Footwear	Fatigue	Footwear* Fatigue
		Pre	Post	Δ	95%CI	Pre	Post	Δ	95%CI			
Ankle	Dorsiflexor	0.09±0.12	0.11±0.12	0.02	-0.06; 0.02	0.04±0.08	0.06±0.11	0.02	-0.05; 0.01	0.002 (1.353)	0.192 (0.536)	0.984 (0.000)
	Plantarflexor	-5.99±2.30	-5.85±1.52	-0.14	-1.16; 0.89	-5.54±1.78	-5.95±2.59	0.41	-0.70; 1.52	0.610 (0.211)	0.654 (0.180)	0.528 (0.255)
	Evertor	0.52±0.51	0.40±0.43	-0.12	-0.07; 0.32	0.47±0.39	0.47±0.56	0.00	-0.19; 0.19	0.952 (0.000)	0.428 (0.320)	0.297 (0.424)
	Invertor	-0.23±0.45	-0.34±0.72	0.11	-0.04; 0.26	-0.27±0.65	-0.31±0.51	0.04	-0.13; 0.21	0.864 (0.063)	0.168 (0.569)	0.563 (0.238)
	Internal rotator	0.36±0.24	0.45±0.29	0.09	-0.19; 0.01	0.39±0.37	0.42±0.35	0.03	-0.13; -0.06	0.992 (0.000)	0.105 (0.674)	0.448 (0.307)
	External rotator	-0.19±0.18	-0.16±0.16	-0.03	-0.07; 0.02	-0.19±0.10	-0.18±0.14	-0.01	-0.06; 0.04	0.714 (0.142)	0.324 (0.403)	0.683 (0.168)
Knee	Extensor	1.62±0.55	1.61±0.60	-0.01	-0.24; 0.26	1.32±0.51	1.37±0.58	0.05	-0.25; 0.16	0.004 (1.253)	0.807 (0.090)	0.728 (0.142)
	Flexor	-4.91±1.56	-5.46±1.69	0.55	-0.27; 1.37	-5.53±2.33	-6.12±2.06	0.59	0.39; 1.56	0.083 (0.721)	0.048 (0.830)	0.958 (0.000)
	Adductor	0.54±0.57	0.58±0.48	0.04	-0.27, 0.21	0.41±0.42	0.41±0.51	0.00	-0.18; 0.18	0.082 (0.724)	0.811 (0.090)	0.820 (0.090)
	Abductor	-0.52±0.68	-0.46±0.81	-0.06	-0.26; 0.14	-0.61±0.69	-0.63±0.78	0.02	-0.21; 0.27	0.169 (0.565)	0.820 (0.090)	0.598 (0.211)
	Internal rotator	0.28±0.18	0.40±0.32	0.12	-0.19; -0.02	0.39±0.35	0.39±0.35	0.00	-0.04, 0.10	0.413 (0.333)	0.042 (0.860)	0.073 (0.749)
	External rotator	-0.17±0.13	-0.19±0.15	0.02	-0.03; 0.08	-0.17±0.11	-0.19±0.14	0.02	-0.65; 0.26	0.999 (0.000)	0.210 (0.514)	0.997 (0.000)
Hip	Flexor	4.89±1.09	4.74±1.05	-0.15	-0.38;0.67	4.00±0.78	4.19±0.87	0.19	-0.70; 1.81	<0.001 (1.787)	0.894 (0.063)	0.319 (0.408)
	Extensor	-8.05±1.81	-9.57±3.18	1.52	0.40; 2.63	-9.79±3.54	-10.34±3.20	0.55	-0.38; 0.25	0.019 (1.000)	0.018 (1.012)	0.245 (0.478)
	Adductor	0.76±0.83	0.64±0.79	-0.12	-0.21; 0.46	0.48±0.65	0.54±0.64	0.06	-0.36; 0.42	0.184 (0.544)	0.816 (0.090)	0.415 (0.333)
	Abductor	-0.86±0.65	-1.01±0.95	0.15	-0.14; 0.45	-1.29±0.92	-1.32±0.99	0.03	-0.11; 0.15	0.014 (1.059)	0.503 (0.271)	0.550 (0.238)
	Internal rotator	0.41±0.26	0.53±0.40	0.12	-0.24; -0.01	0.63±0.43	0.61±0.41	-0.02	-0.08; 0.07	0.033 (0.899)	0.267 (0.454)	0.082 (0.255)
	External rotator	-0.27±0.17	-0.22±0.12	-0.05	-0.13; -0.02	-0.27±0.14	-0.27±0.14	0.00	-1.01; 0.11	0.402 (0.339)	0.286 (0.434)	0.326 (0.403)

For kinetic data, we observed a significant main effect of “Fatigue” for knee flexor moment, knee internal rotator moment, and hip extensor moment (p<0.05; d = 0.83–1.01) (Table 2). Pair-wise comparisons showed a significant fatigue-related increase in peak knee flexor moment (p<0.05; d = 0.83; 0.02 N.m/kg), peak knee internal rotation moment (p<0.05; d = 0.86; 0.06 N.m/kg), and peak hip extensor moment (p<0.02; d = 1.01; 1.26 N.m/kg). Moreover, the statistical analysis did not yield any significant footwear by fatigue interactions for joint moments (Table 2).

For peak positive hip power in sagittal and frontal planes and peak negative hip power in horizontal plane, we observed significant main effects of “footwear” (p<0.03; d = 0.92–1.06). (Table 3). Pairwise comparisons revealed that peak positive hip power in sagittal plane (p<0.03; d = 0.98; 2.39 w/kg), peak positive hip power in frontal plane (p<0.02; d = 1.06; 0.54 w/kg), and peak negative hip power in horizontal plane (p<0.03; d = 0.92; 0.43 w/kg) were greater with anti-pronation shoes. Also, the statistical analyses indicated a significant main effect of “Fatigue” for peak negative ankle power in the sagittal plane (p<0.01; d = 1.25). Pair-wise comparisons showed a significant fatigue-related increase in peak negative ankle joint power in the sagittal plane (p<0.01; d = 1.25; 2.00 w/kg) (Table 3). Finally, the statistical analysis did not yield any significant footwear by fatigue interactions for joint powers (Table 3).

**Table 3:** Data are means and standard deviations for footwear-specific peak joint powers (W/kg) in sagittal, frontal and horizontal planes during running in fatigued and unfatigued condition.

Joint	Plan	Sign	Neutral shoe				Anti-pronation shoe					Footwear	Fatigue	Footwear* Fatigue
			Pre	Post	Δ	95%CI	Pre	Post	Δ	95%CI				
**Ankle**	Sagittal	Positive	18.99±9.84	16.12±6.77	-2.87	-0.46; 6.19	15.89±6.75	16.16±9.01	0.27	-4.48; 4.04	0.267 (0.454)	0.191 (0.536)	0.337 (0.392)
		Negative	-12.18±4.00	-13.90±4.70	1.72	-0.49; 3.91	-12.00±4.30	-14.29±6.70	2.29	-0.34; 4.92	0.892 (0.063)	0.004 (1.250)	0.773 (0.110)
	Frontal	Positive	1.76±1.82	1.67±2.71	-0.09	-0.59; 0.79	1.39±1.00	1.29±1.07	-0.10	-0.46; 0.68	0.324 (0.403)	0.686 (0.168)	0.965 (0.000)
		Negative	-0.90±0.67	-0.88±0.86	-0.02	-0.34; 0.31	-0.99±0.81	-0.95±0.61	-0.04	-0.38; 0.30	0.397 (0.346)	0.782 (0.110)	0.931 (0.000)
	Horizontal	Positive	0.62±0.65	0.45±0.34	-0.17	-0.13; 0.45	0.49±0.37	0.47±0.36	-0.02	-0.12; 0.17	0.420 (0.327)	0.295 (0.429)	0.355 (0.375)
		Negative	-0.26±0.24	-0.23±0.45	-0.03	-0.22; 0.16	-0.16±0.26	-0.23±0.51	0.07	-0.12; 0.27	0.337 (0.392)	0.779 (0.110)	0.360 (0.375)
**Knee**	Sagittal	Positive	19.53±9.32	25.70±12.15	6.17	-11.95; -0.38	25.29±12.27	26.98±12.59	1.69	-7.96; 4.57	0.108 (0.667)	0.091 (0.703)	0.249 (0.375)
		Negative	-5.33±4.70	-4.88±3.15	-0.45	-2.78; 1.39	-4.55±3.81	-5.14±4.13	0.59	-1.68; 2.87	0.713 (0.155)	0.899 (0.063)	0.591 (0.220)
	Frontal	Positive	0.46±0.46	0.55±0.57	0.09	-0.32, 0.14	0.58±0.95	0.56±0.79	-0.02	-0.37, 0.41	0.478 (0.286)	0.755 (0.063)	0.637 (0.191)
		Negative	-0.37±0.37	-0.55±0.80	0.18	-0.06; 0.40	-0.77±1.15	-0.62±0.61	-0.15	-0.66; 0.36	0.133 (0.621)	0.943 (0.000)	0.219 (0.550)
	Horizontal	Positive	0.37±0.43	0.57±1.05	0.20	-0.53; 0.11	0.56±1.00	0.84±1.66	0.28	-0.61; 0.04	0.164 (0.574)	0.109 (0.663)	0.520 (0.263)
		Negative	-0.48±0.38	-0.38±0.33	-0.10	-0.25; 0.06	-0.42±0.35	-0.46±0.62	0.04	-0.15; 0.22	0.917 (0.000)	0.679 (0.168)	0.157 (0.078)
**Hip**	Sagittal	Positive	18.74±9.40	20.48±9.17	1.74	-4.01;0.53	22.03±9.65	21.96±11.17	-0.07	-2.98; 3.11	0.026 (0.947)	0.272 (0.434)	0.407 (0.582)
		Negative	-17.26±6.65	-15.38±5.00	-1.88	-4.87; 1.12	-14.50±5.83	-15.86±6.98	1.36	-1.18; 3.90	0.272 (0.663)	0.813 (0.090)	0.059 (0.790)
	Frontal	Positive	1.09±0.96	1.00±0.89	-0.09	-0.25; 0.43	1.65±0.86	1.54±1.02	-0.11	-0.31; 0.53	0.014 (1.059)	0.457 (0.300)	0.947 (0.000)
		Negative	-1.25±0.90	-1.38±0.93	0.13	-0.36; 0.61	-1.23±1.13	-1.06±0.74	-0.17	-0.65; 0.30	0.356 (0.449)	0.869 (0.063)	0.408 (0.339)
	Horizontal	Positive	0.39±0.51	0.28±0.25	-0.11	-0.07; 0.28	0.44±0.33	0.35±0.34	-0.09	-0.06; 0.25	0.381 (0.358)	0.123 (0.637)	0.923 (0.000)
		Negative	-0.47±0.52	-0.62±0.77	0.15	-0.09; 0.40	-0.96±1.41	-1.00±1.31	0.04	-0.34; 0.41	0.029 (0.924)	0.436 (0.314)	0.532 (0.2555)

## Discussion

This study is the first to examine lower limb joint angles, moments and powers of female recreational runners when running in unfatigued and fatigued condition with anti-pronation versus neutral shoes.

The main findings of this study were i) smaller peak ankle eversion angles when running with anti-pronation shoes compared with neutral shoes, ii) lower peak positive hip power in sagittal plane, peak positive hip power in frontal plane, and peak negative hip power in horizontal plane in anti-pronation vs neutral shoes, iii) fatigue-related increases in peak ankle eversion angle, irrespective of the used footwear, iv) fatigue-induced increases in peak knee flexor moment, peak knee internal rotation moment, peak hip extensor moment, and peak negative ankle joint power, irrespective of the used footwear, v) no significant footwear by fatigue interaction effects for all measures of joint kinetics and kinematics.

All our participants showed clinical signs of over-pronation and our study findings revealed larger rearfoot angles during running in neutral compared with anti-pronation shoes. The anti-pronation shoe reduced peak rearfoot eversion by approximately 3°. Results of this study (i.e., lower peak eversion angles during running in anti-pronation compared with neutral shoes) are in accordance with findings from previous studies [19, 26] despite methodological differences in the experimental setup (e.g., shoe design, running speed) of these studies. Also, the existing studies found increased tibial internal rotation, increased knee internal rotation, and decreased knee adduction with fatigue [35, 36]. Of note, an increased peak ankle eversion angle is associated with local fatigue of the ankle encompassing muscles. Even though the observed 3° increase in peak eversion angle appears neglectable, its cumulative effects during cyclic activities like running are clinically relevant and a potential contributing factor to injury [15]. The observed fatigue-related increase in peak ankle eversion over the course of the running trial indicates that runners may require additional pronation support in fatigued condition. The effects of fatigue on foot alignment should therefore be considered when selecting appropriate running footwear [37]. Notably, we observed a reduction in peak rearfoot eversion angle when running in anti-pronation compared with neutral shoes.

This study is the first that observed significantly smaller peak ankle dorsiflexor moments, peak knee extensor moments, and peak hip flexor moments as well as larger peak hip extensor moments when running with anti-pronation versus neutral shoes. Given that no comparable studies are available in the literature on lower limb joint moments and how they are affected by fatigue and anti-pronation shoes, our findings are difficult to interpret. Of note, analyses of joint moments provide clinically relevant information on joint loading and muscle functioning during running [38]. There is evidence that greater knee adduction moments are associated with higher medial load distributions within the knee joint [39] which may result in the progression of osteoarthritis. Previously, we were able to show that measures of joint mechanical power discriminate between healthy and pathological gait patterns [40]. In this regard, we observed that positive joint power is associated with high joint loads [40], while negative joint power is associated with shock absorption and load dissipation [40]. Also, this is the first study that observed significant fatigue-related increases in peak knee internal rotation moment, peak hip extensor moment, and peak hip internal rotator moment. There is evidence that fatigue induces performance declines due to peripheral changes at the level of the muscle and/or due to insufficient drive of the central nervous system to the motor neurons [1]. It can be hypothesized that large knee adduction moments may increase the risk of overload to medial knee structures which again contributes to iliotibial band friction [41], and patellofemoral pain syndromes [42] in runners. In the present study, both anti-pronation shoes and muscle fatigue did not change the peak knee adduction moment during the stance phase of a running cycle. With regards to the effects of fatigue on knee adduction moments during walking and running, there are conflicting findings in the literature [43, 44]. For example, Murdock et al. reported an increased knee adduction moment [44], while Walter et al. demonstrated a reduction in knee adduction moment [43] after the application of a muscular fatigue protocol. In this study, we could not find an effect of fatigue on knee adduction moment during running. This is in agreement with a study of Longpre and co-workers who induced lower limb fatigue using resistance exercises for the knee flexors/extensors at 50% of maximum voluntary isometric contraction [45]. These authors could not find any fatigue-related changes in knee adduction moment during running. However, it has to be noted that there are only few studies available that examined the impact of fatigue on knee mechanics during running. Therefore, our study adds substantial contributions to this field of research by reporting fatigue-related changes for female runners with overpronation when running in neutral versus anti-pronation shoes.

Our results revealed that peak positive hip power in sagittal and frontal planes and peak negative hip power in horizontal plane were greater when running with the anti-pronation compared with the neutral shoe, irrespective of the fatigue condition. There is evidence that positive joint powers are associated with higher joint loads [46]. Therefore, greater peak positive hip power in sagittal and frontal planes during running with anti-pronation shoes could possibly induce greater hip joint loads. Moreover, we observed significant fatigue-related increases in peak negative ankle joint power in the sagittal plane, irrespective of the used footwear. In this regards, we noted that at the time of initial ground contact, the ankle was within a few degrees from the neutral dorsi-/plantar flexion position [47]. After initial ground contact, ankle dorsiflexor muscles contract eccentrically thereby absorbing power (negative power). This again allows the foot to be lowered gently to the ground [48]. Adequate functioning of the neuromuscular system (i.e., m. quadriceps) during this phase of running could help protect the musculoskeletal system from potentially adverse impulsive loading. It seems that the quadriceps femoris activity contributes to energy absorption at the initial heel contact to reduce ground reaction force [49]. Of note, the quadriceps muscle is eccentrically contracted (negative knee joint power) during the initial ground contact which may enable weight acceptance, particularly because quadriceps muscle dissipates most of the energy at heel contact [50]. However, we could not detect any main effects of footwear for negative power of the knee joint.

This study has some limitations, we only tested female runners, and therefore the findings may not be applicable to male runners given that female runners demonstrate different lower extremity movement patterns during running [51]. Another study limitation is that we did not assess the long-term effects of anti-pronation footwear which is why this needs to be verified in future studies. Finally, we did not compare the effects of different types of anti-pronation shoes which is why this should be examined in future studies.

## Conclusions

Running with anti-pronation compared with neutral shoes produced lower peak moments and powers in lower limb joints that enable runners to better control rear foot eversion. The observed fatigue-related increase in peak ankle eversion over the course of the running trial indicates that runners may require additional pronation support in the fatigued condition. Given that physical fatigue increased peak moments and powers in lower limb joints during running, fatigue related effects on running biomechanics should be considered when selecting appropriate running footwear. Overall, the anti-pronation shoes used in the present study could possibly improve rearfoot eversion and lower limb joint moments in female runners with pronated feet during both unfatigued and fatigued conditions. Of note, our results revealed that peak positive hip power in sagittal and frontal planes and peak negative hip power in horizontal plane were greater when running with the anti-pronation compared with the neutral shoe, irrespective of the experimental condition (fatigued versus non-fatigued).

## Supporting information

S1 File Log files of the statistical analyses. (DOCX)

## References

[1] Gandevia SC. Spinal and supraspinal factors in human muscle fatigue. Physiol Rev. 2001; 81(4): 1725–89. 10.1152/physrev.2001.81.4.1725 11581501

[2] Anbarian M, Esmaeili H. Effects of running-induced fatigue on plantar pressure distribution in novice runners with different foot types. Gait Posture. 2016; 48:52–6. 10.1016/j.gaitpost.2016.04.029 27477708

[3] Fourchet F, Kelly L, Horobeanu C, Loepelt H, Taiar R, Millet G. High-intensity running and plantar-flexor fatigability and plantar-pressure distribution in adolescent runners. J Athl Train. 2015; 50(2): 117–25. 10.4085/1062-6050-49.3.90 25531143 PMC4495434

[4] Mizrahi J, Verbitsky O, Isakov E. Shock accelerations and attenuation in downhill and level running. Clin Biomech. 2000; 15(1): 15–20. 10.1016/s0268-0033(99)00033-9 10590340

[5] Radzak KN, Putnam AM, Tamura K, Hetzler RK, Stickley CD. Asymmetry between lower limbs during rested and fatigued state running gait in healthy individuals. Gait Posture. 2017; 51: 268–74. 10.1016/j.gaitpost.2016.11.005 27842295

[6] Weist R, Eils E, Rosenbaum D. The influence of muscle fatigue on electromyogram and plantar pressure patterns as an explanation for the incidence of metatarsal stress fractures. Am J Sports Med. 2004; 32(8): 1893–8. 15572318 10.1177/0363546504265191

[7] Van Gent R, Siem D, van Middelkoop M, Van Os A, Bierma-Zeinstra S, Koes B. Incidence and determinants of lower extremity running injuries in long distance runners: a systematic review. Brit J Sport Med. 2007; 41(8): 469–80. 10.1136/bjsm.2006.033548 PMC2465455 17473005

[8] Hulme A, Nielsen RO, Timpka T, Verhagen E, Finch C. Risk and protective factors for middle-and long-distance running-related injury. Sports Med. 2017; 47(5): 869–86. 10.1007/s40279-016-0636-4 27785775

[9] Tong JW, Kong PW. Association between foot type and lower extremity injuries: systematic literature review with meta-analysis. J Orthop Sports Phys Ther. 2013; 43(10): 700–14. 10.2519/jospt.2013.4225 23756327

[10] Farahpour N, Jafarnezhadgero A, Allard P, Majlesi M. Muscle activity and kinetics of lower limbs during walking in pronated feet individuals with and without low back pain. J Electromyogr Kinesiol. 2018; 39: 35–41. 10.1016/j.jelekin.2018.01.006 29413451

[11] Chuter VH, de Jonge XAJ. Proximal and distal contributions to lower extremity injury: a review of the literature. Gait Posture. 2012; 36(1): 7–15. 10.1016/j.gaitpost.2012.02.001 22440758

[12] Raissi GRD, Cherati ADS, Mansoori KD, Razi MD. The relationship between lower extremity alignment and Medial Tibial Stress Syndrome among non-professional athletes. BMC Sports Sci Med Rehabil. 2009; 1(1): 11. 10.1186/1758-2555-1-11 PMC2700791 19519909

[13] Ryan M, Grau S, Krauss I, Maiwald C, Taunton J, Horstmann T. Kinematic analysis of runners with achilles mid-portion tendinopathy. Foot Ankle Int. 2009; 30(12): 1190–5. 10.3113/FAI.2009.1190 20003878

[14] Banwell HA, Mackintosh S, Thewlis D. Foot orthoses for adults with flexible pes planus: a systematic review. J Foot Ankle Res. 2014; 7(1): 23 10.1186/1757-1146-7-23 24708560 PMC4108129

[15] Hreljac A. Etiology, prevention, and early intervention of overuse injuries in runners: a biomechanical perspective. Phys Med Rehabil Clin. 2005; 16(3): 651–67. 10.1016/j.pmr.2005.02.002 16005398

[16] Cheung R, Ng G. Efficacy of motion control shoes in exhausted runners: a 3-dimensional kinematics analysis. J Sci Med Sport. 2005; 8(1): 176.

[17] Clarke T, Frederick E, Hamill C. The effects of shoe design parameters on rearfoot control in running. Med Sci Sport Exer. 1983; 15(5): 376–81. 6645865

[18] Rose A, Birch I, Kuisma R. Effect of motion control running shoes compared with neutral shoes on tibial rotation during running. Physiotherapy. 2011; 97(3): 250–5. 10.1016/j.physio.2010.08.013 21820544

[19] Cheung RT, Ng GY. Efficacy of motion control shoes for reducing excessive rearfoot motion in fatigued runners. Phys Ther Sport. 2007; 8(2): 75–81.

[20] Napier C, Willy RW. Logical fallacies in the running shoe debate: let the evidence guide prescription. BMJ Publishing Group Ltd and British Association of Sport and Exercise Medicine; 2018. 10.1136/bjsports-2018-100117 30352861

[21] Jenkins WL, Williams DB III, Williams K, Hefner J, Welch H. Sex differences in total frontal plane knee movement and velocity during a functional single-leg landing. Phys Ther Sport. 2017; 24: 1–6. 10.1016/j.ptsp.2016.09.005 28013024

[22] Redmond AC, Crosbie J, Ouvrier RA. Development and validation of a novel rating system for scoring standing foot posture: the Foot Posture Index. Clin Biomech. 2006; 21(1): 89–98. 10.1016/j.clinbiomech.2005.08.002 16182419

[23] Shih Y, Lin K-L, Shiang T-Y. Is the foot striking pattern more important than barefoot or shod conditions in running? Gait Posture. 2013; 38(3): 490–4. 10.1016/j.gaitpost.2013.01.030 23507028

[24] Cavanagh PR, Lafortune MA. Ground reaction forces in distance running. J Biomech. 1980; 13(5): 397–406. 7400169 10.1016/0021-9290(80)90033-0

[25] Redmond A. The foot posture index: easy quantification of standing foot posture: six item version: FPI-6: user guide and manual. United Kingdom. 2005: 1–19.

[26] Stacoff A, Nigg BM, Reinschmidt C, van den Bogert AJ, Lundberg A. Tibiocalcaneal kinematics of barefoot versus shod running. J Biomech. 2000; 33(11): 1387–95. 10940397 10.1016/s0021-9290(00)00116-0

[27] Cappozzo A, Catani F, Della Croce U, Leardini A. Position and orientation in space of bones during movement: anatomical frame definition and determination. Clin Biomech. 1995; 10(4): 171–8. 10.1016/0268-0033(95)91394-t 11415549

[28] Davis RB III, Ounpuu S, Tyburski D, Gage JR. A gait analysis data collection and reduction technique. Hum Movement Sci. 1991; 10(5): 575–87.

[29] Besier TF, Sturnieks DL, Alderson JA, Lloyd DG. Repeatability of gait data using a functional hip joint centre and a mean helical knee axis. J Biomech. 2003; 36(8): 1159–68. 12831742 10.1016/s0021-9290(03)00087-3

[30] Dempster WT, Gabel WC, Felts WJ. The anthropometry of the manual work space for the seated subject. Am J Phys Anthropol. 1959; 17(4): 289–317. 13815872 10.1002/ajpa.1330170405

[31] Borg G. Borg's perceived exertion and pain scales: Human kinetics; 1998.

[32] Medicine ACoS. ACSM's health-related physical fitness assessment manual: Lippincott Williams & Wilkins; 2013.

[33] Koblbauer IF, van Schooten KS, Verhagen EA, van Dieën JH. Kinematic changes during running-induced fatigue and relations with core endurance in novice runners. J Sci Med Sport. 2014; 17(4): 419–24. 10.1016/j.jsams.2013.05.013 23790535

[34] Cohen J. Statistical power analysis for the behavioral sciences. 2nd Hillsdale, NJ: erlbaum; 1988.

[35] Dierks TA, Davis IS, Hamill J. The effects of running in an exerted state on lower extremity kinematics and joint timing. J Biomech. 2010; 43(15): 2993–8. 10.1016/j.jbiomech.2010.07.001 20663506

[36] Dierks TA, Manal KT, Hamill J, Davis I. Lower extremity kinematics in runners with patellofemoral pain during a prolonged run. Med Sci Sports Exerc. 2011; 43(4): 693–700. 10.1249/MSS.0b013e3181f744f5 20798656

[37] Riley PO, Dicharry J, Franz J, Della Croce U, Wilder RP, Kerrigan DC. A kinematics and kinetic comparison of overground and treadmill running. Med Sci Sports Exerc. 2008; 40(6): 1093–100. 10.1249/MSS.0b013e3181677530 18460996

[38] Jafarnezhadgero AA, Shad MM, Majlesi M. Effect of foot orthoses on the medial longitudinal arch in children with flexible flatfoot deformity: A three-dimensional moment analysis. Gait Posture. 2017; 55: 75–80. 10.1016/j.gaitpost.2017.04.011 28419877

[39] Jafarnezhadgero AA, Oliveira AS, Mousavi SH, Madadi-Shad M. Combining valgus knee brace and lateral foot wedges reduces external forces and moments in osteoarthritis patients. Gait Posture. 2018; 59: 104–10. 10.1016/j.gaitpost.2017.09.040 29028621

[40] Jafarnezhadgero A, Shad MM, Majlesi M, Zago M. Effect of kinesio taping on lower limb joint powers in individuals with genu varum. J Bodyw Mov Ther. 2018; 22(2): 511–8. 10.1016/j.jbmt.2017.06.009 29861259

[41] Andriacchi T, editor The biomechanics of running and knee injuries. Symposium on Sports Medicine: the Knee; 1985: CV Mosby.

[42] Stefanyshyn D. Footwear traction and knee joint moments. J Biomech. 2006; 39: S181.

[43] Walter JP, D'lima DD, Colwell Jr CW, Fregly BJ. Decreased knee adduction moment does not guarantee decreased medial contact force during gait. J Orthop Res. 2010; 28(10): 1348–54. 10.1002/jor.21142 20839320 PMC2984615

[44] Murdock GH, Hubley-Kozey CL. Effect of a high intensity quadriceps fatigue protocol on knee joint mechanics and muscle activation during gait in young adults. Eur J Appl Physiol. 2012; 112(2): 439–49. 10.1007/s00421-011-1990-4 21573776

[45] Longpré HS, Potvin JR, Maly MR. Biomechanical changes at the knee after lower limb fatigue in healthy young women. Clin Biomech. 2013; 28(4): 441–7. 10.1016/j.clinbiomech.2013.02.010 23528628

[46] Hendershot BD, Wolf EJ. Mediolateral joint powers at the low back among persons with unilateral transfemoral amputation. Arch Phys Med Rehabil. 2015; 96(1): 154–7. 10.1016/j.apmr.2014.07.402 25102386

[47] Nicola TL, Jewison DJ. The anatomy and biomechanics of running. Clin Sports Med. 2012; 31(2): 187–201. 10.1016/j.csm.2011.10.001 22341011

[48] Rose J, Gamble JG. Human walking. Lippincott Williams & Wilkins Philadelphia USA 2006: 111–4.

[49] Liikavainio T, Isolehto J, Helminen HJ, Perttunen J, Lepola V, Kiviranta I, et al Loading and gait symmetry during level and stair walking in asymptomatic subjects with knee osteoarthritis: importance of quadriceps femoris in reducing impact force during heel strike? Knee. 2007; 14(3): 231–8. 10.1016/j.knee.2007.03.001 17451958

[50] Barbieri F, Gobbi L, Lee Y, Pijnappels M, van Dieën J. Effect of triceps surae and quadriceps muscle fatigue on the mechanics of landing in stepping down in ongoing gait. Ergonomics. 2014; 57(6): 934–42. 10.1080/00140139.2014.903302 24697241

[51] Ferber R, Davis IM, Williams Iii DS. Gender differences in lower extremity mechanics during running. Clin Biomech. 2003; 18(4): 350–7. 10.1016/s0268-0033(03)00025-1 12689785

