# Supplementary figures and images for: Effects of anti-pronation shoes on lower limb kinematics and kinetics in female runners with pronated feet: The role of physical fatigue
Source: PLoS One. 2019 May 14;14(5):e0216818. doi: 10.1371/journal.pone.0216818 (PMC6516670; doi:10.1371/journal.pone.0216818)

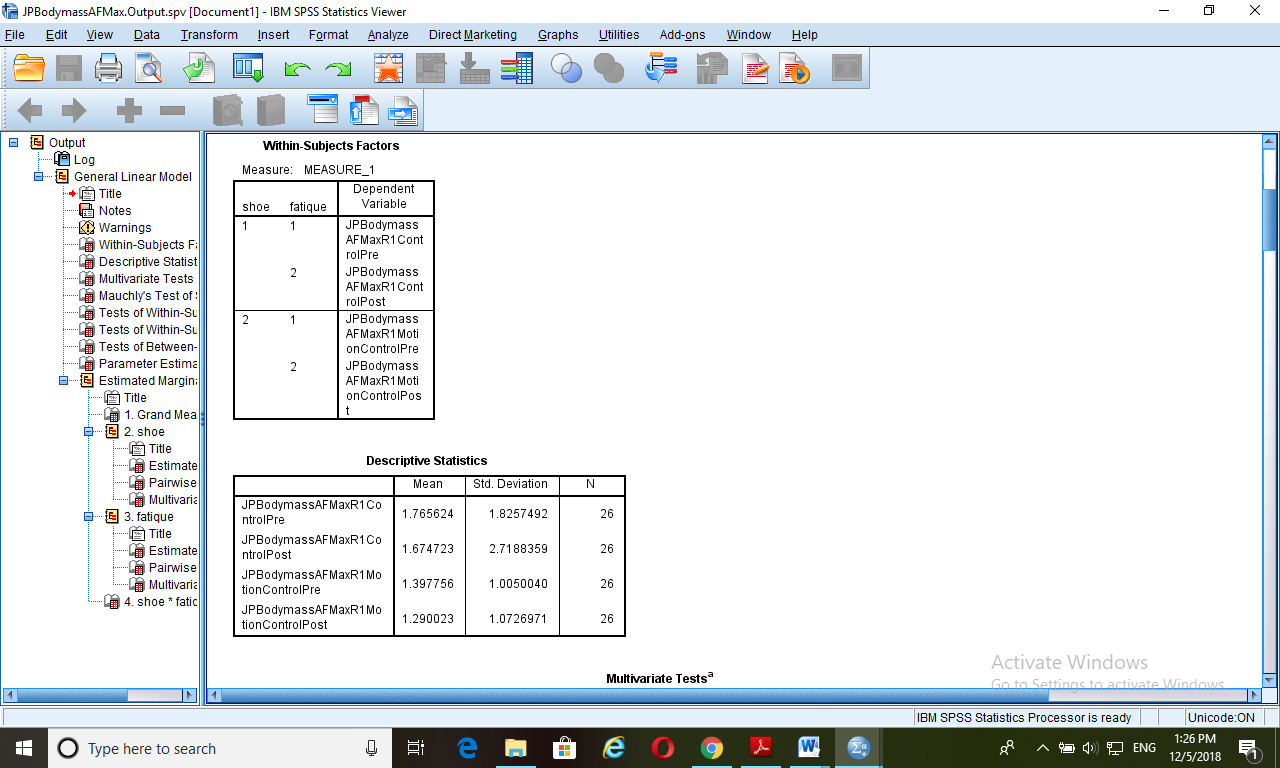


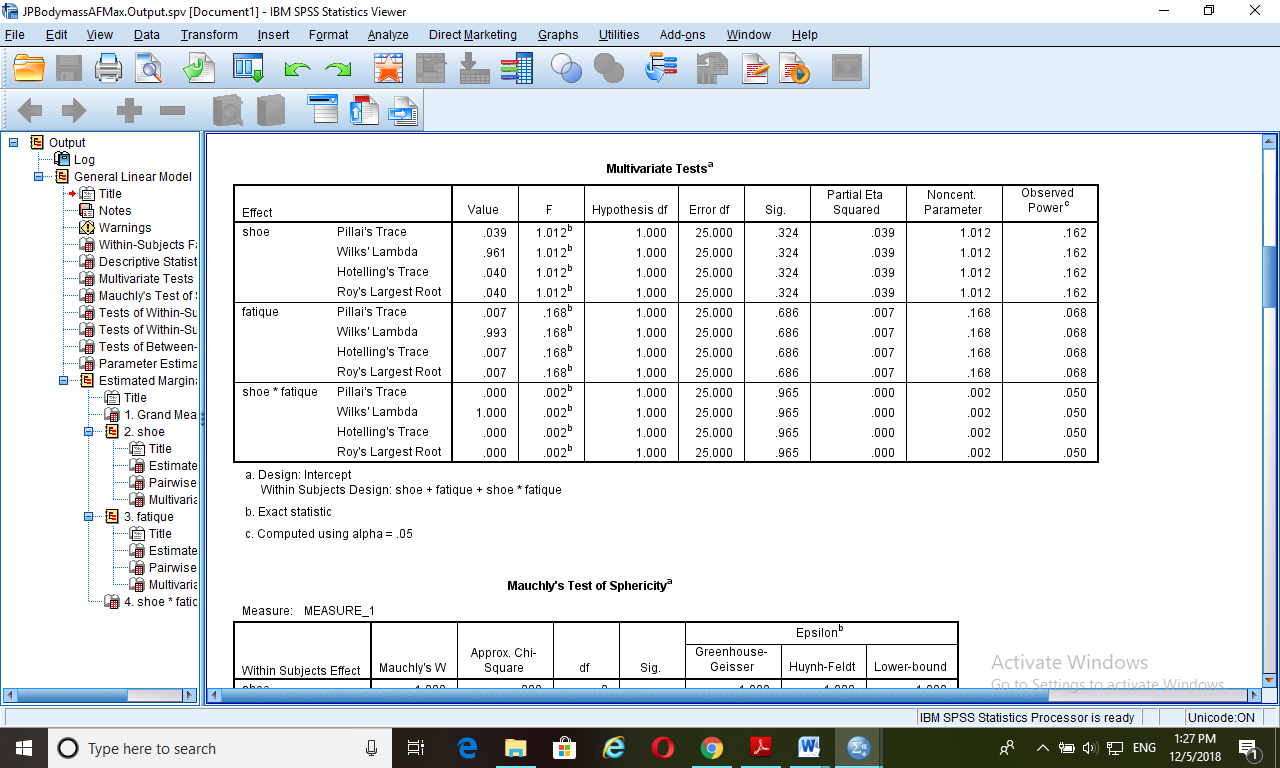


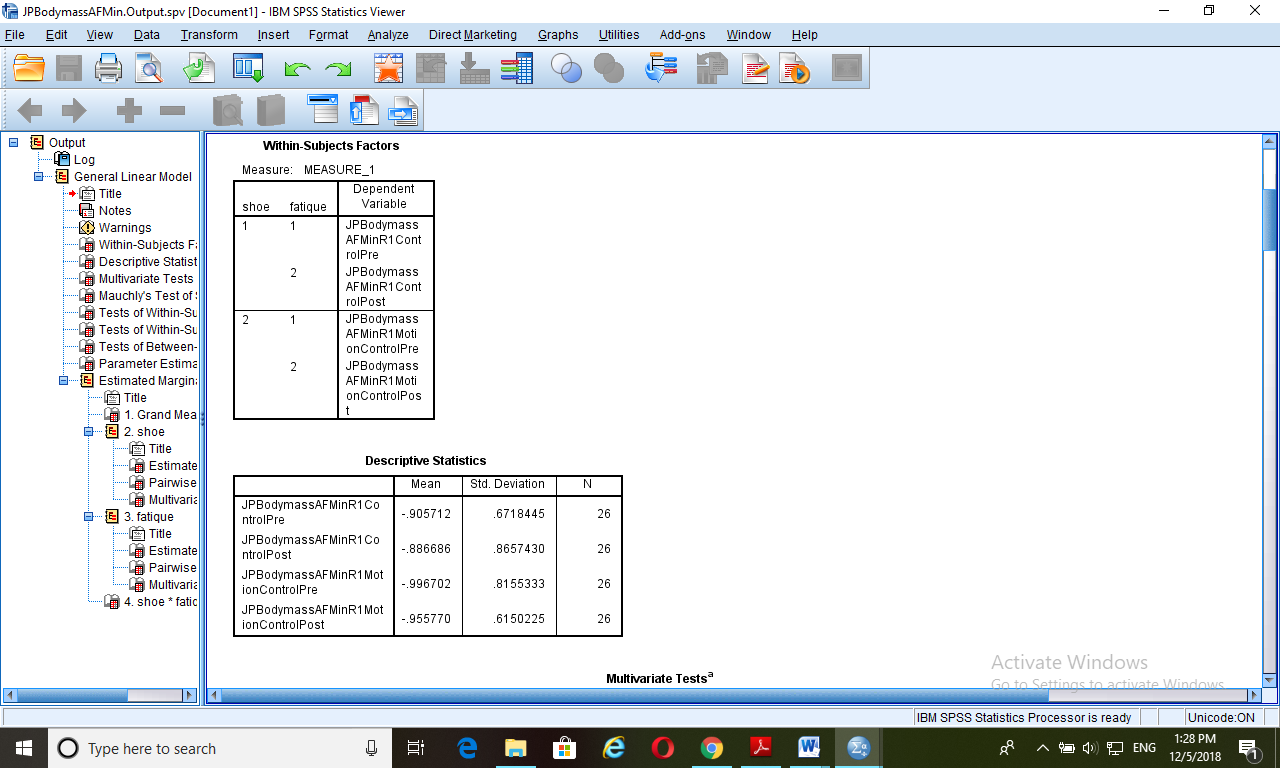


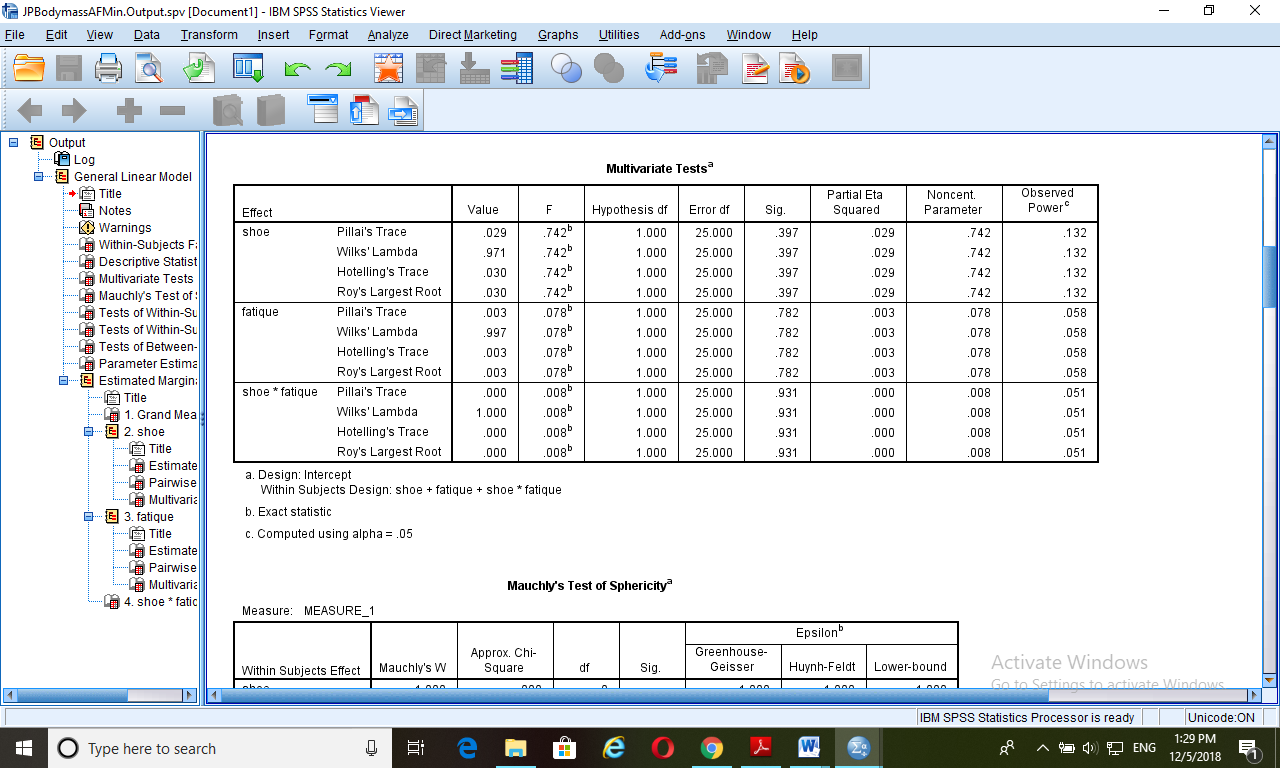


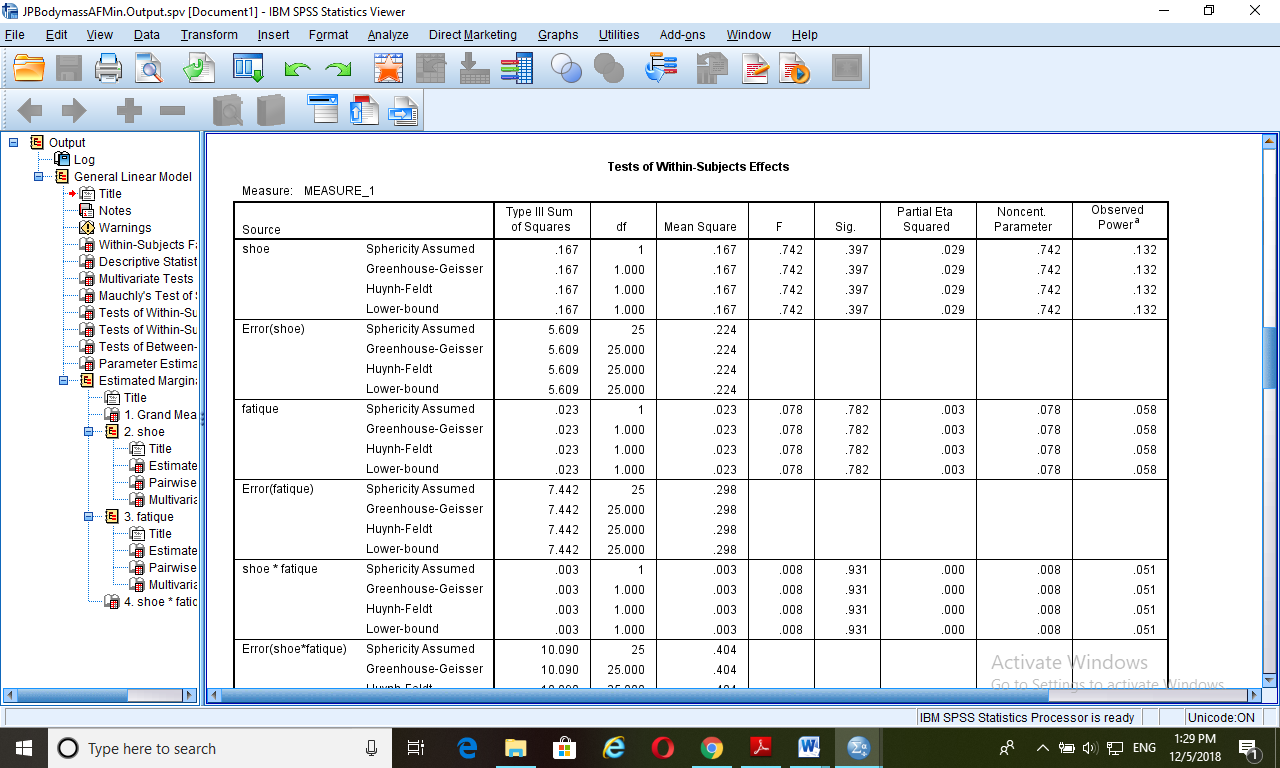


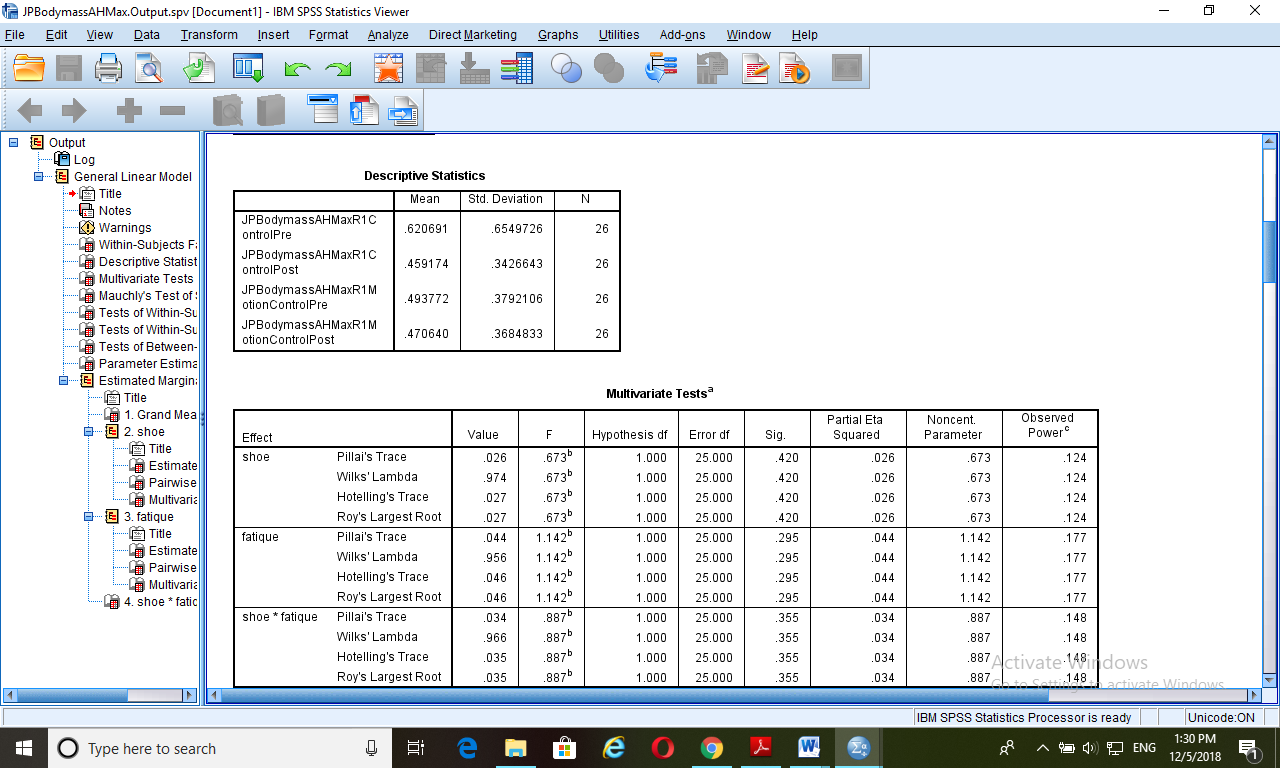


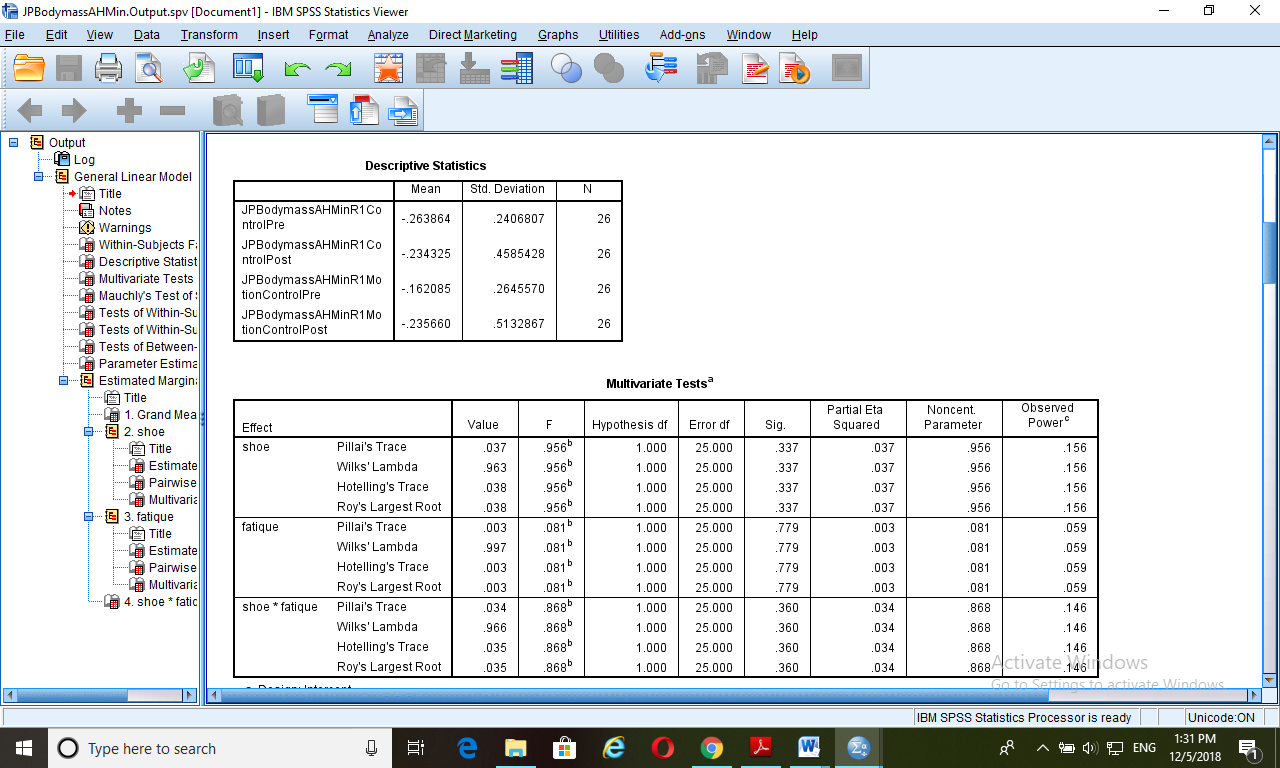


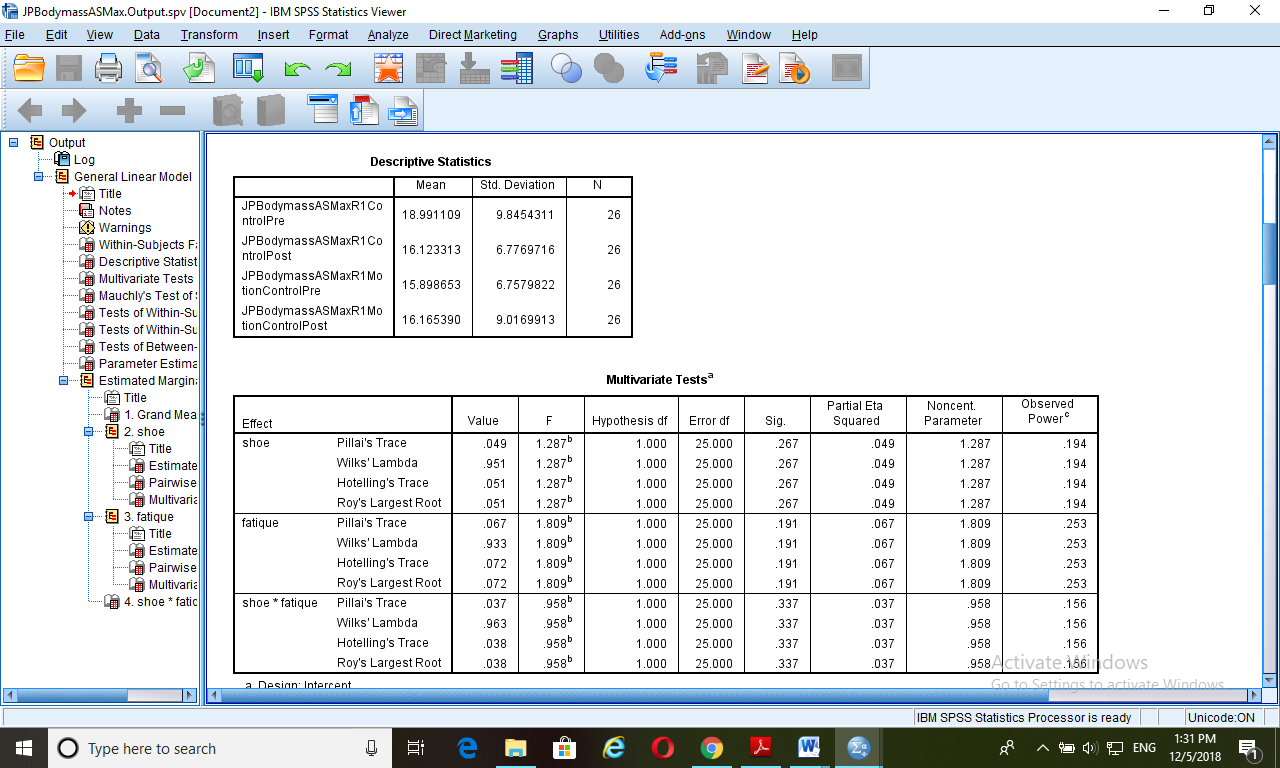


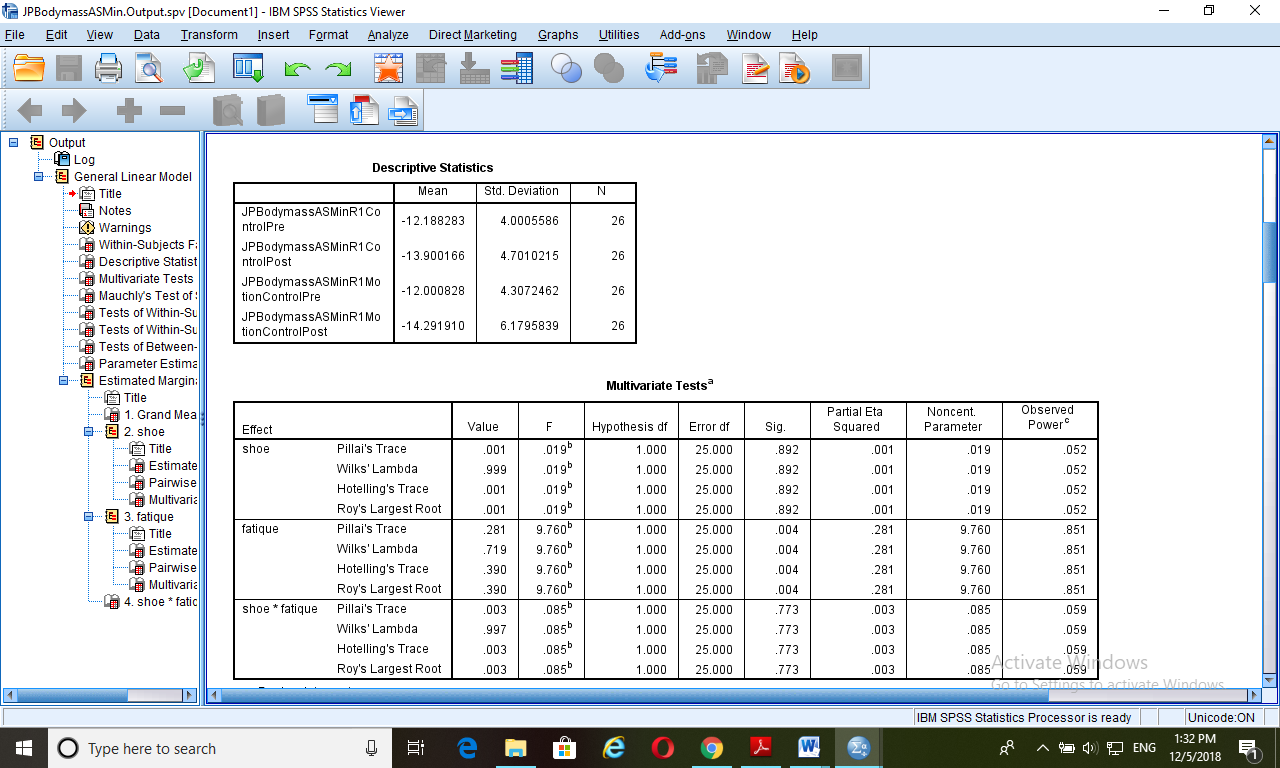


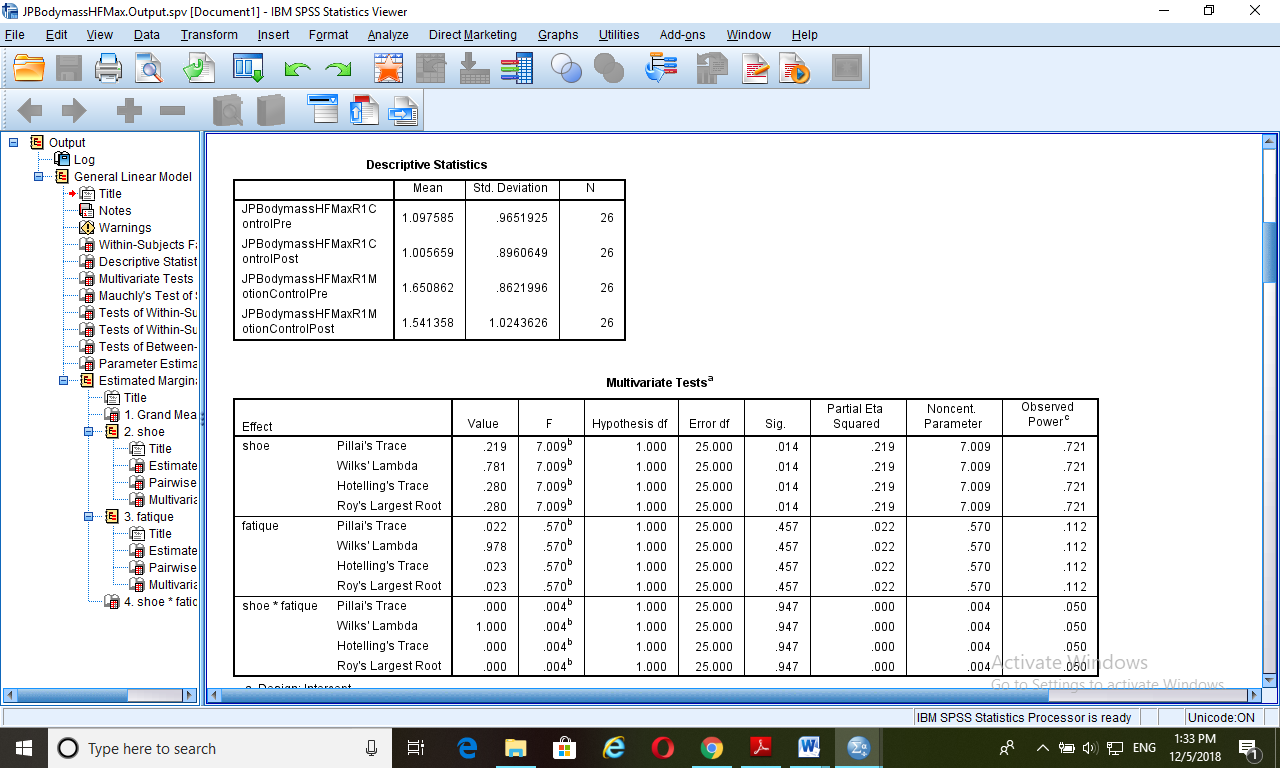


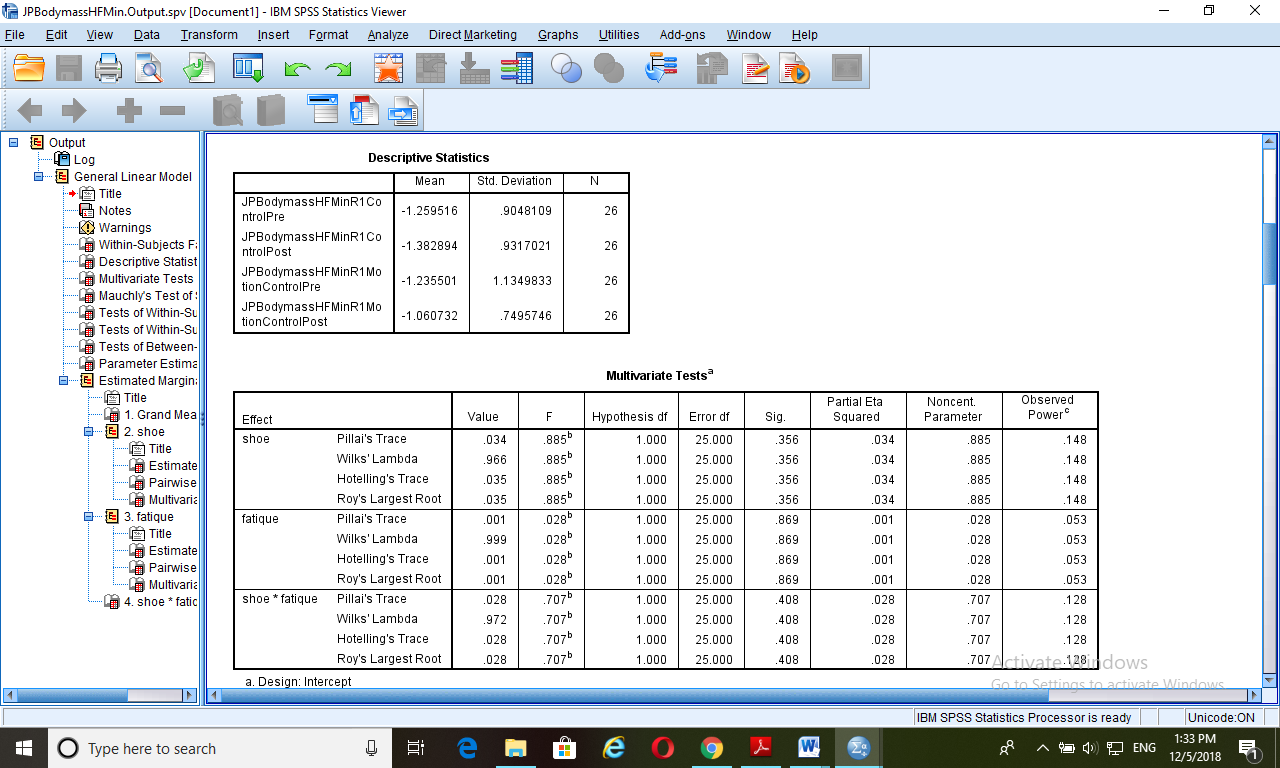


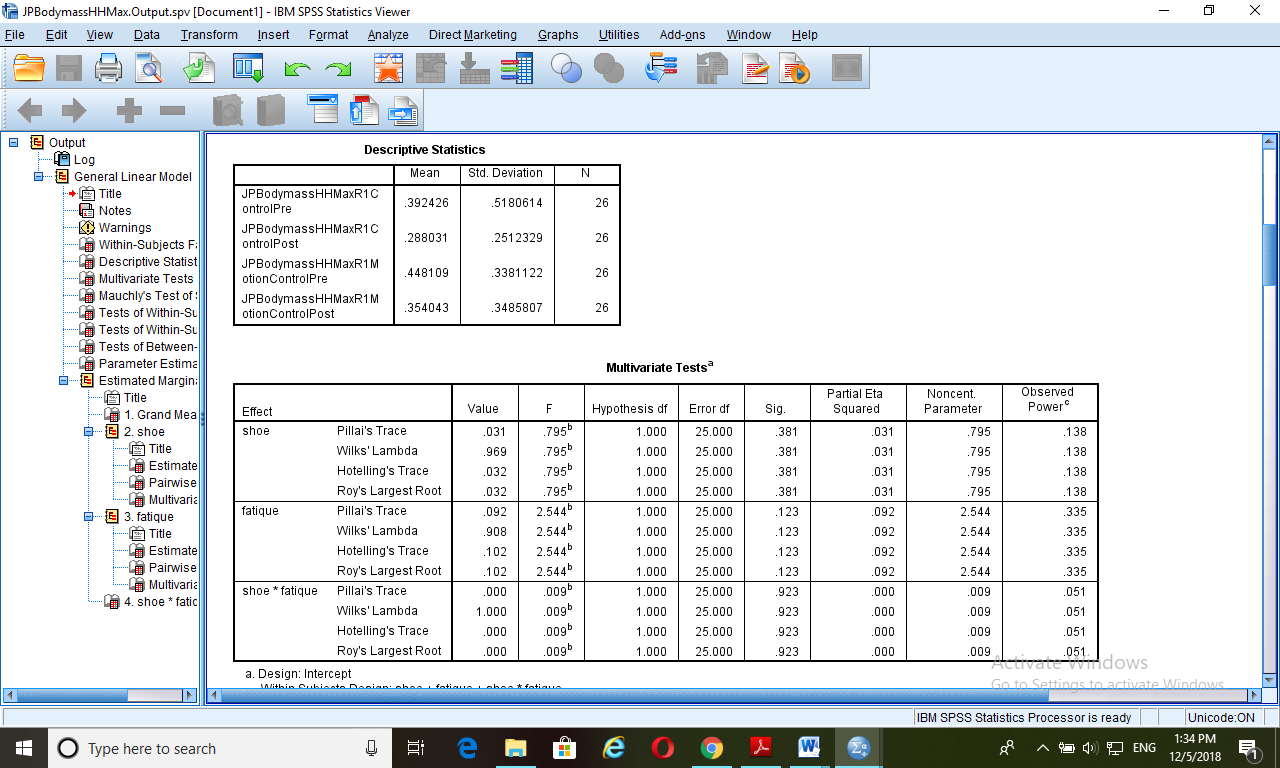


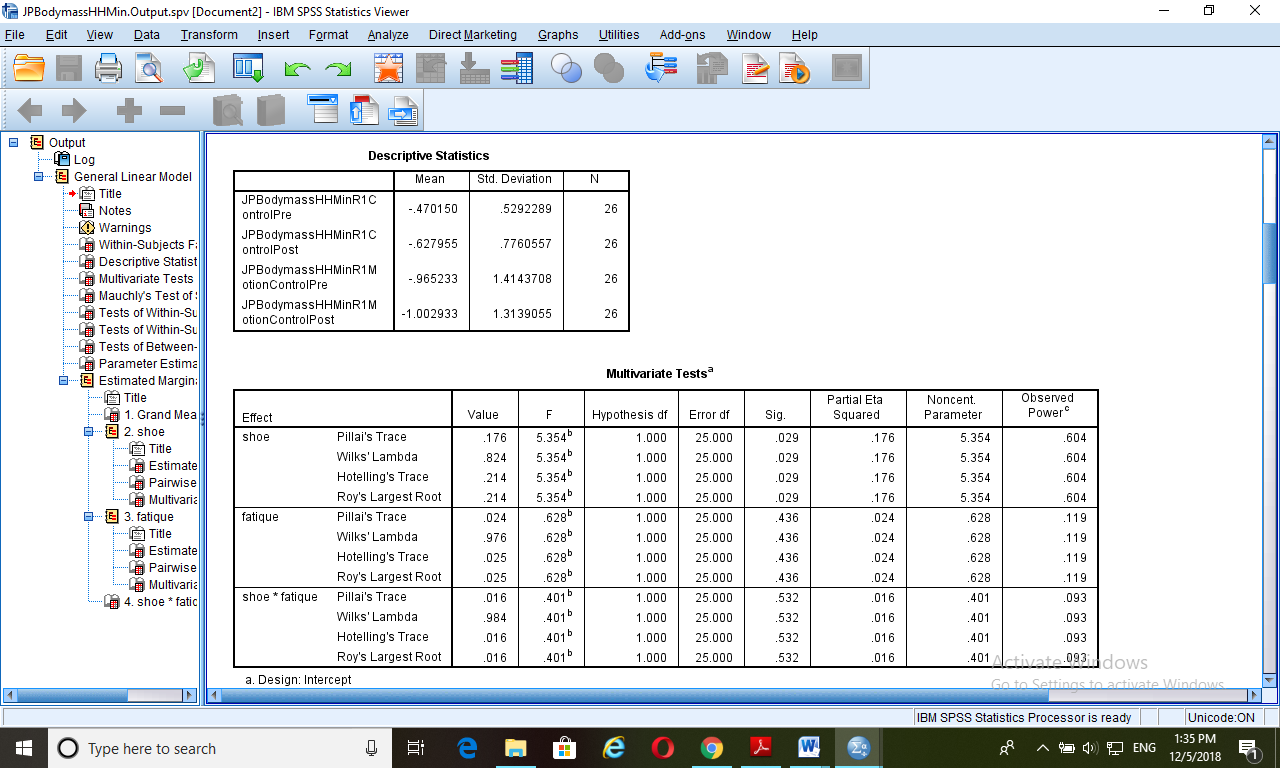


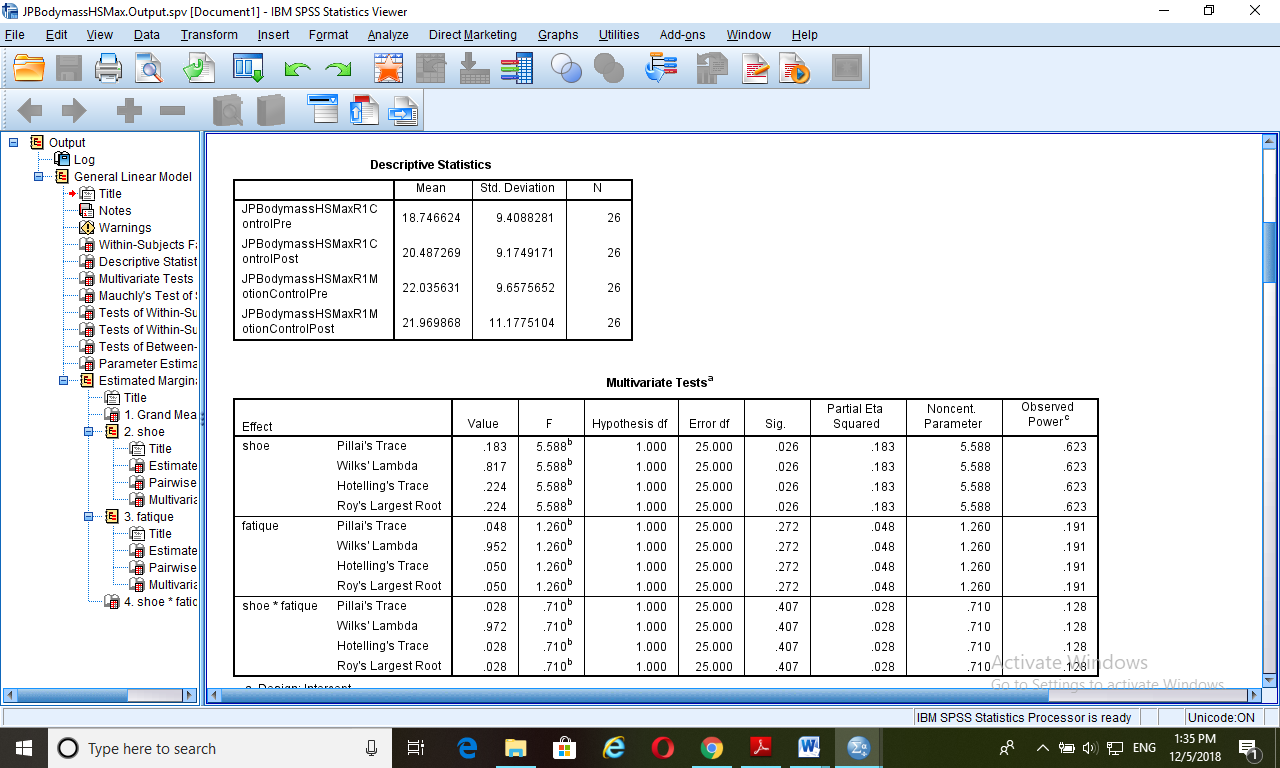


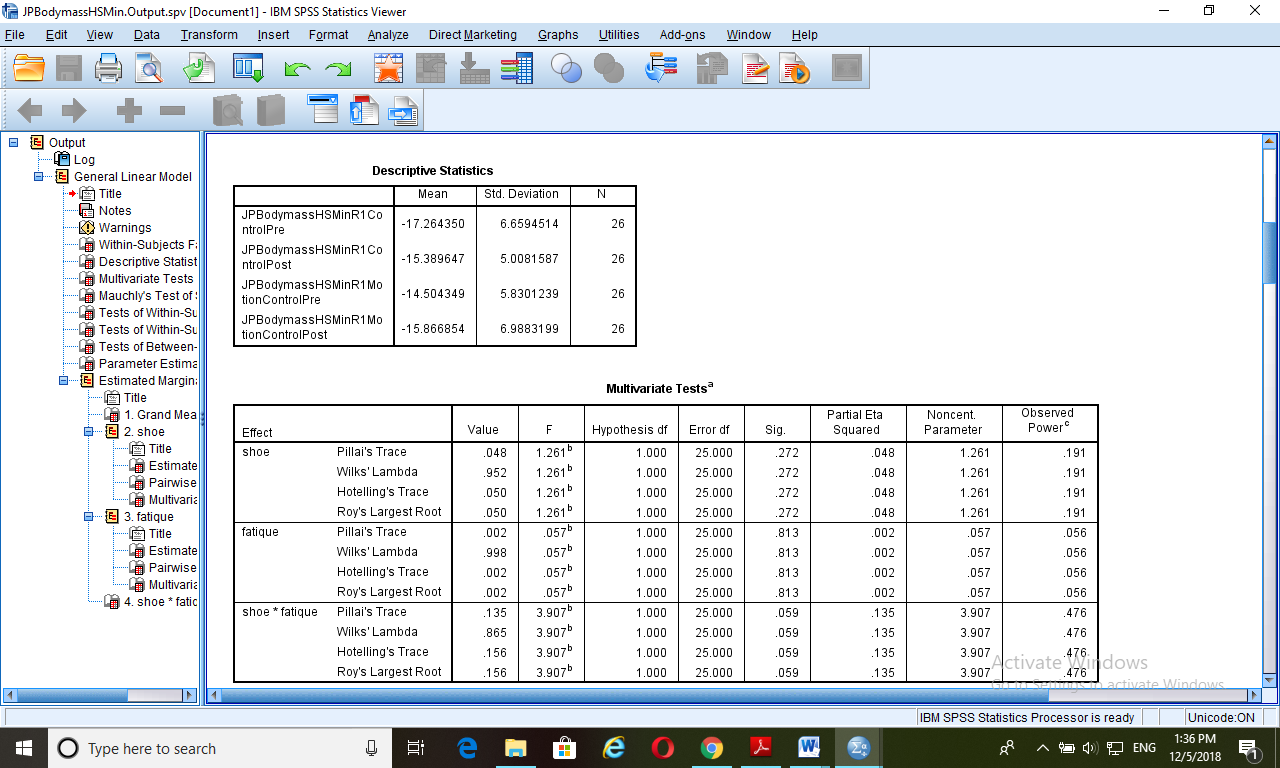


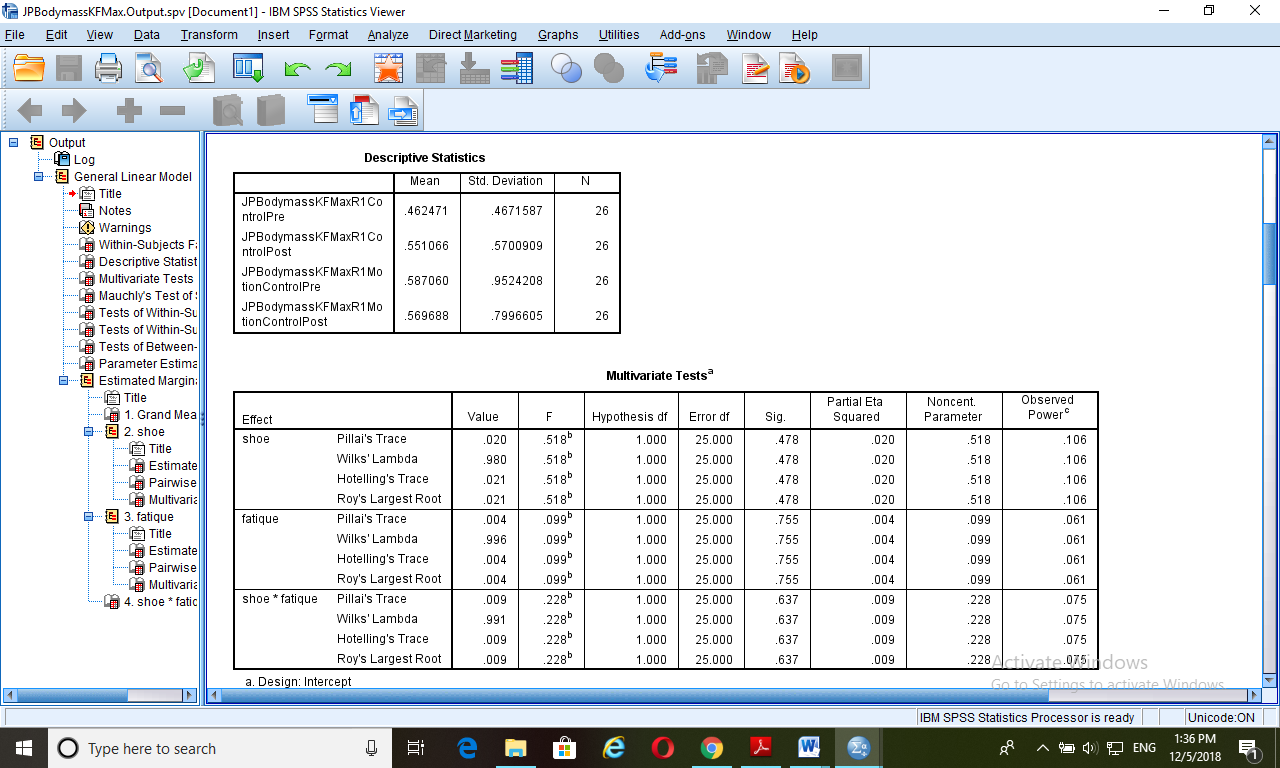


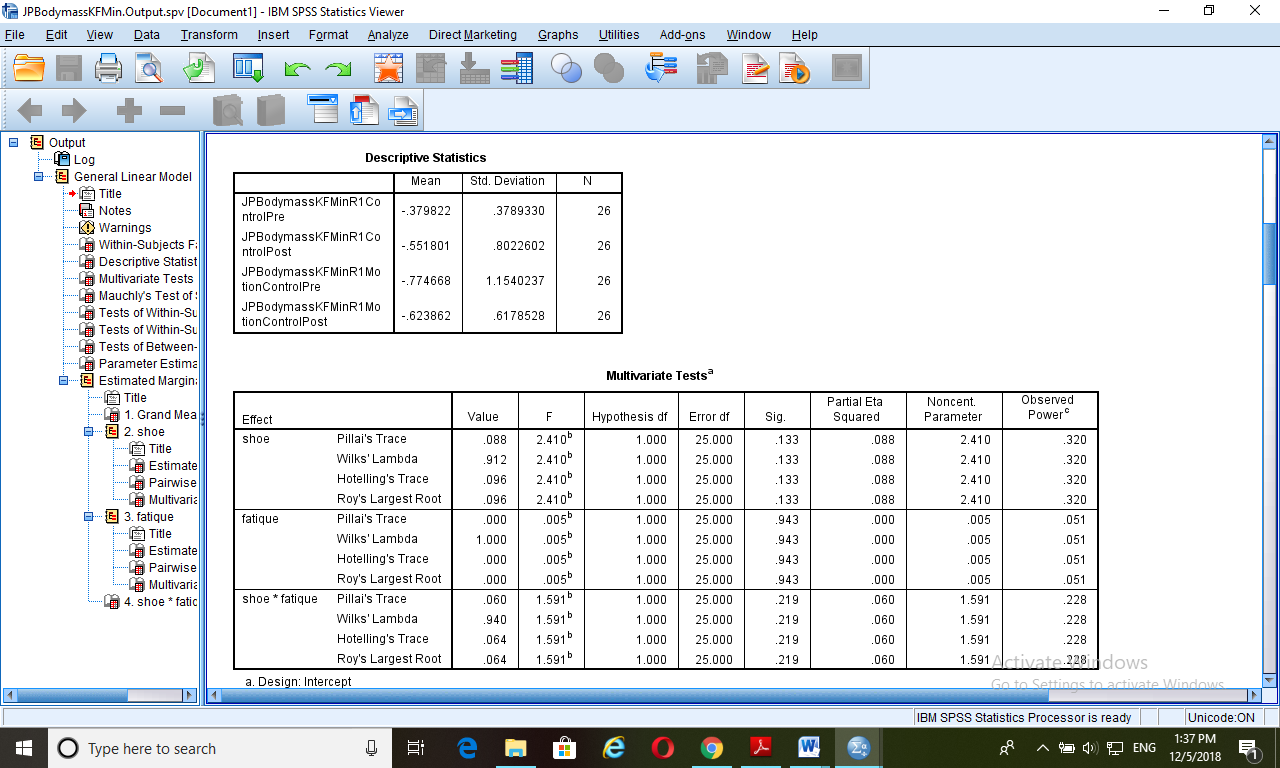


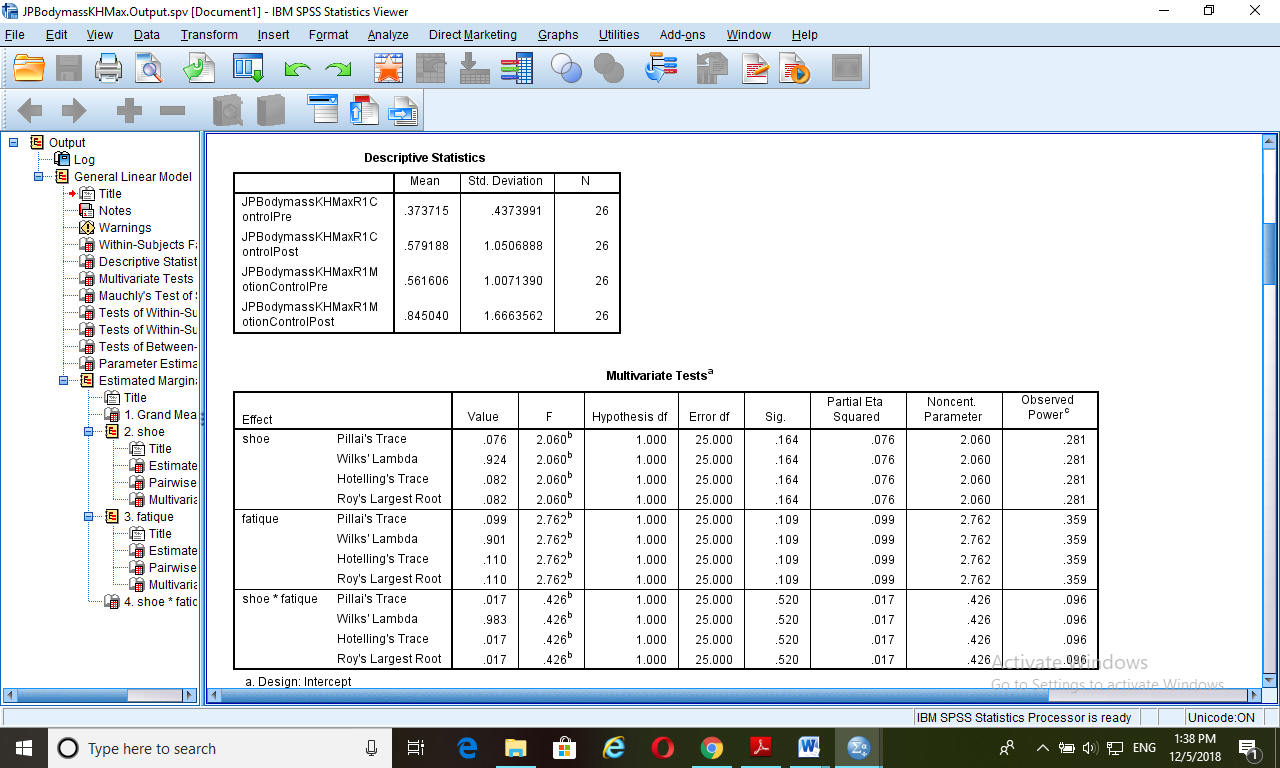


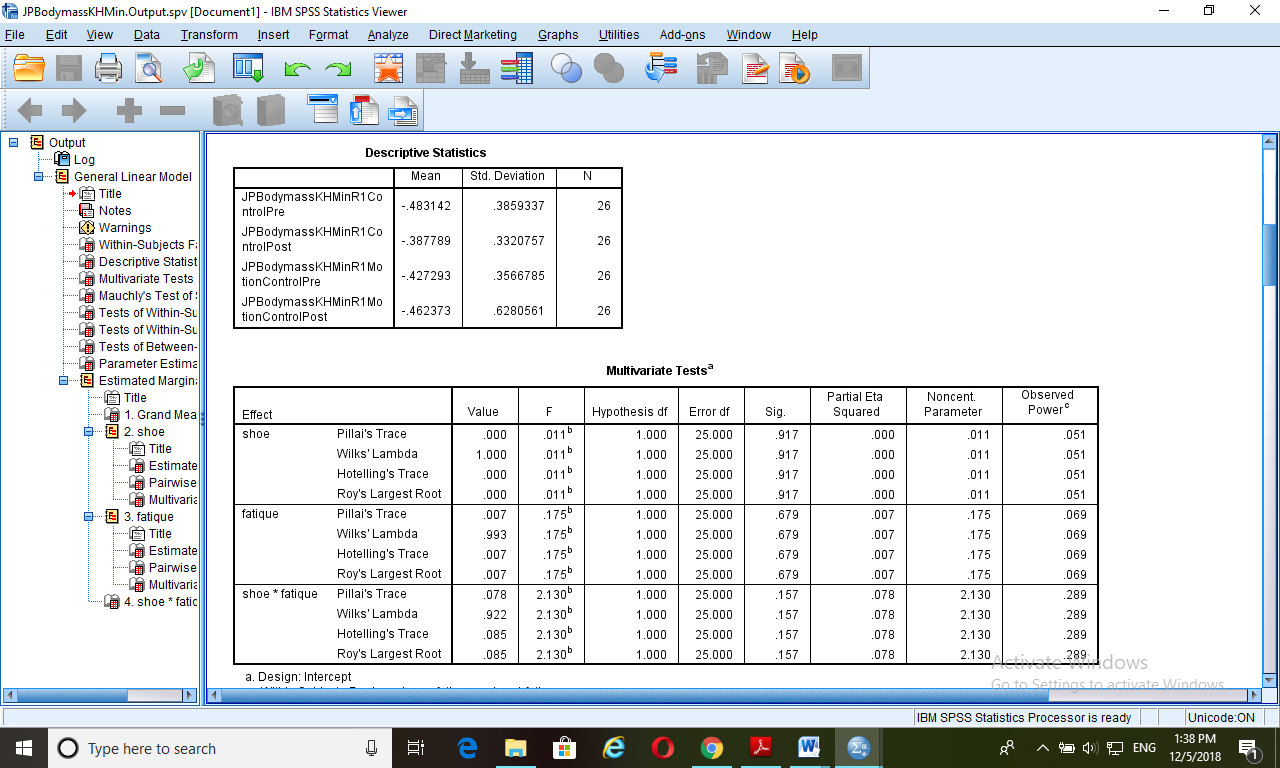


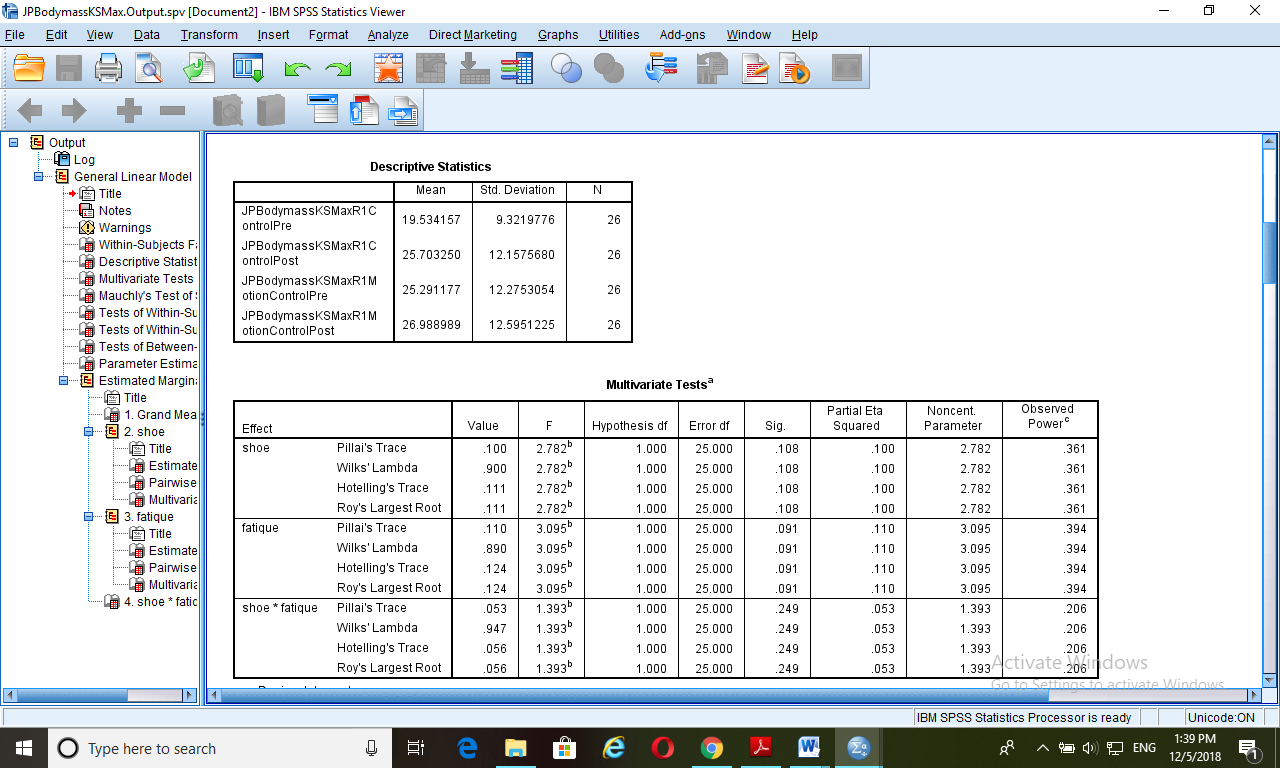


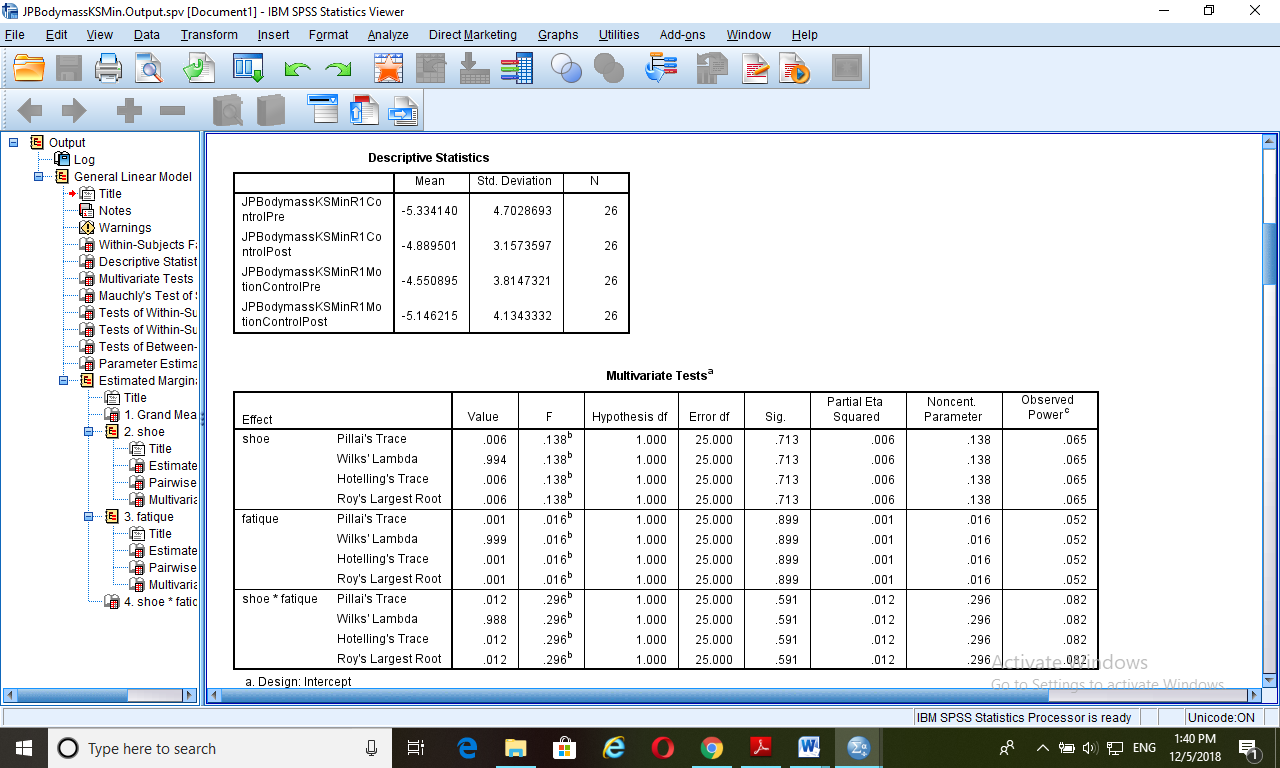

Supplement: S1 File — (DOCX) [file pone.0216818.s001.docx]
